# Targeting the FOSL1/IKKα positive feedback loop attenuates glioblastoma malignancy *via* suppression of NF-κB signaling

**DOI:** 10.7150/thno.121770

**Published:** 2026-01-01

**Authors:** Beichen Zhang, Hao Tian, Haoyu Zhou, Yichang Wang, Ke Gao, Yiyang Cao, Mingjing Zhou, Maode Wang, Wei Wu, Jia Wang

**Affiliations:** 1 Department of Neurosurgery, The First Affiliated Hospital of Xi'an Jiaotong University, Xi'an, Shaanxi 710061, China.; 2 Center for Brain Science, The First Affiliated Hospital of Xi'an Jiaotong University, Xi'an, Shaanxi 710061, China.; These authors contributed equally: Beichen Zhang, Hao Tian, Haoyu Zhou.

**Keywords:** FOSL1/IKKα positive feedback loop, NF-κB signaling, glioblastoma, malignancy, nano-based siRNA delivery system

## Abstract

**Rationale:** Glioblastoma (GBM), the most aggressive primary tumor of the central nervous system, remains clinically intractable because of marked molecular heterogeneity and persistent therapeutic resistance, underscoring the need for novel targeted interventions.

**Methods:** Gene expression profiles from the TCGA and CGGA datasets were analyzed to identify prognostic transcription factors. Functional validation was performed using lentiviral-mediated knockdown and overexpression in GBM cell lines, followed by assays for proliferation, migration, invasion and apoptosis. Underlying molecular mechanisms were investigated using chromatin immunoprecipitation (ChIP), co-immunoprecipitation (co-IP), ubiquitination assays, and *in vitro* kinase assays. A nanocapsule-based siRNA delivery system was engineered and evaluated for its stability, cellular uptake, and blood-brain barrier penetration. Therapeutic efficacy was assessed in orthotopic GBM models using bioluminescence imaging, survival analysis, and histopathological examination.

**Results:** This study identified FOS-like antigen 1 (FOSL1) as a key oncogenic driver that facilitates GBM progression through a positive feedback loop with inhibitor of nuclear factor kappa-B kinase subunit alpha (*IKKα*). Mechanistic studies revealed that FOSL1 enhances transcriptional upregulation of *IKKα*, while *IKKα* reciprocally stabilizes FOSL1 by suppressing its phosphorylation and subsequent ubiquitin-proteasomal degradation. Ubiquitination assays further identified ubiquitin C-terminal hydrolase L3 (UCHL3) as the principal de-ubiquitinase mediating FOSL1 stabilization through selective removal of K48-linked polyubiquitin chains. This FOSL1-driven positive feedback loop ultimately activated NF-κB signaling, resulting in enhanced invasion and malignancy of GBM. From a therapeutic standpoint, targeting the FOSL1/IKKα/UCHL3 feedback axis yielded significant attenuation of multiple malignant phenotypes of GBM using a novel nanoparticle-based siRNA delivery system (plofsome@*siFOSL1*), which effectively suppressed *FOSL1* expression.

**Conclusions:** The findings of this study establish a previously unrecognized FOSL1/IKKα/UCHL3 positive feedback loop as a central driver of GBM pathogenesis through activation of NF-κB signaling, providing a promising molecular target for future GBM therapeutic strategies.

## Introduction

Glioblastoma (GBM) is the most aggressive and lethal primary tumor of the central nervous system, with a median survival of less than 16 months 1-6. The inherent heterogeneity of GBM generates diverse epigenetic signatures and complex transcriptional regulation, thereby driving a wide spectrum of malignant biological behaviors including immune evasion, therapy resistance, and rapid progression. Despite current treatment strategies, including surgery, chemotherapy, and radiation, the survival of patients with GBM remains dismal, with a nearly inevitable recurrence occurring at a mean time of 6.9 months 7, 8. Therefore, elucidating the molecular mechanisms governing GBM pathogenesis and tumorigenesis is critical for identifying novel therapeutic targets and improving patient prognosis 9-12.

Transcription factors (TFs) are pivotal regulators of gene expression networks that orchestrate essential cellular processes and play critical roles in malignant tumor progression 13-16. As evidenced by recent research, TFs exhibit prognostic significance in diverse malignancies, with dysregulated expression patterns demonstrating strong correlations with clinical outcomes across multiple cancer subtypes 13, 17. FOS-like antigen 1 (FOSL1), a member of the AP-1 transcription factor family, contains a basic leucine zipper (bZIP) domain that enables dimerization with JUN proteins and DNA binding to AP-1 consensus motifs 18-22. Under physiological conditions, *FOSL1* plays crucial roles in placental development, osteoblast lineage differentiation, and skeletal morphogenesis. Previous studies have confirmed low *FOSL1* expression in normal tissues, whereas its expression is frequently upregulated in tumorigenic processes such as GBM 19. Accumulating evidence has demonstrated that *FOSL1* is essential for GBM pathogenesis, as it transcriptionally regulates downstream effector genes and drives multiple malignant processes including stemness maintenance, angiogenesis, and epithelial-to-mesenchymal transition (EMT), thereby promoting tumor recurrence, invasion, and therapeutic resistance 31, 32. Nevertheless, the precise transcriptional mechanisms through which *FOSL1* coordinates oncogenic programs remain incompletely understood.

The nuclear factor kappa-light-chain-enhancer of activated B cells (NF-κB) signaling pathway plays a pivotal role in diverse physiological and pathological processes 28. A critical step in canonical NF-κB activation involves the phosphorylation-dependent activation of the inhibitor of nuclear factor kappa-B kinase (IKK) complex, composed of IKKα, IKKβ, and IKKγ (also known as NEMO) subunits. This complex is recruited and activated by upstream signals such as TNFα and IL-1, and is widely considered a principal regulator of NF-κB signaling 29. Subsequently, inhibitor of kappa B (IκB) proteins undergo phosphorylation and degradation, leading to the release of NF-κB dimers that translocate into the nucleus to drive the transcription of target genes 30. Activation of NF-κB signaling promotes GBM cell proliferation, survival, angiogenesis, and invasion, thereby contributing to tumor progression 23-27. Nevertheless, the upstream mechanisms responsible for activating the NF-κB pathway in GBM cells remain incompletely understood. In this study, elevated *FOSL1* expression was shown to promote GBM progression by transcriptionally upregulating *IKKα*, while IKKα-mediated phosphorylation stabilizes FOSL1 by inhibiting its ubiquitin-proteasomal degradation. The de-ubiquitinase ubiquitin C-terminal hydrolase L3 (UCHL3) functions as a key regulator by selectively cleaving K48-linked polyubiquitin chains on FOSL1. Collectively, the FOSL1/IKKα/UCHL3 regulatory axis forms a positive feedback loop that sustains NF-κB signaling activation, thereby driving GBM progression.

To explore the therapeutic potential of disrupting the FOSL1/IKKα feedback loop, a stable and efficient nano-based siRNA delivery system, plofsome@*siFOSL1*, was engineered to enable lysosomal escape and blood-brain barrier penetration. In orthotopic GBM models, plofsome@*siFOSL1*-mediated *FOSL1* knockdown significantly suppressed tumor growth and extended survival.

Taken together, these findings indicate that a FOSL1-dependent IKKα positive feedback mechanism underlies the malignant characteristics of GBM and highlight its potential as a therapeutic target. Based on this rationale, this study systematically dissects the upstream and downstream regulators of FOSL1 and evaluates the therapeutic potential of its targeted suppression via a nano-based delivery platform, thereby defining a novel FOSL1-centric oncogenic axis in GBM.

## Results

### Elevated FOSL1 expression is associated with poor prognosis in GBM patients

To identify TFs most strongly associated with prognosis in GBM, mRNA expression profiles were obtained from the TCGA and CGGA datasets and subjected to univariate Cox regression analysis. This approach yielded 2,009 and 3,990 candidate genes, respectively (Figure S1A-B). These candidate gene lists were subsequently intersected with the TRRUST 33 dataset, resulting in the identification of 20 TFs showing the strongest correlation with GBM prognosis (Figure S1C). Non-negative matrix factorization (NMF) clustering analysis was then applied to segregate GBM samples into two subgroups with distinct survival outcomes (Figure S1D-E), which was further supported by Kaplan-Meier analysis (Figure S1F-G) and principal components analysis (PCA) (Figure S1H-I). Taken together, these findings underscore the prognostic relevance of TF expression in GBM heterogeneity, indicating the utility of TF-based profiling for patient stratification.

Differential expression analysis of TCGA and CGGA cohorts was subsequently conducted to identify prognostically critical TFs among the stratified subgroups (Figure S2A-B). Intersection analysis revealed 4 TFs that were significantly upregulated and 5 that were significantly downregulated in GBM samples (Figure S2C). Among these, multivariate Cox regression identified *FOSL1* as a prominent prognostic indicator (Figure S2D-E). Elevated *FOSL1* expression showed a pronounced inverse correlation with patient survival, supporting its function as both a compelling prognostic biomarker and a pathogenic driver in GBM.

To further characterize the clinical relevance of *FOSL1* expression in glioma, TCGA and CGGA datasets were evaluated to determine *FOSL1* distribution across glioma subtypes. *FOSL1* expression increased with ascending WHO tumor grade and was particularly enriched in GBM relative to lower-grade gliomas (Figure S3A-B). Immunoblot analysis of resected GBM tissues further confirmed markedly elevated FOSL1 levels compared to matched paracancerous tissues (Figure S3C). Correspondingly, multiple GBM cell lines exhibited substantially elevated FOSL1 expression compared with normal human astrocytes (Figure S3D-E). Moreover, immunohistochemical (IHC) staining of samples collected from patients who underwent surgical resection at the Department of Neurosurgery, The First Affiliated Hospital of Xi'an Jiaotong University (2016-2021), demonstrated pronounced FOSL1 overexpression in GBM tissue (Figure S3F). Kaplan-Meier survival analysis revealed that high *FOSL1* expression corresponded with significantly shortened overall survival in GBM patients (Figure S3G), with comparable trends observed in both TCGA and CGGA datasets (Figure S3H-K). Collectively, these data establish upregulated *FOSL1* expression as a robust indicator of adverse prognosis in glioma, particularly in GBM, and underscore its potential as a clinically relevant diagnostic and prognostic biomarker.

### FOSL1 is functionally required for multiple malignant behaviors in GBM

**Figure 1:**
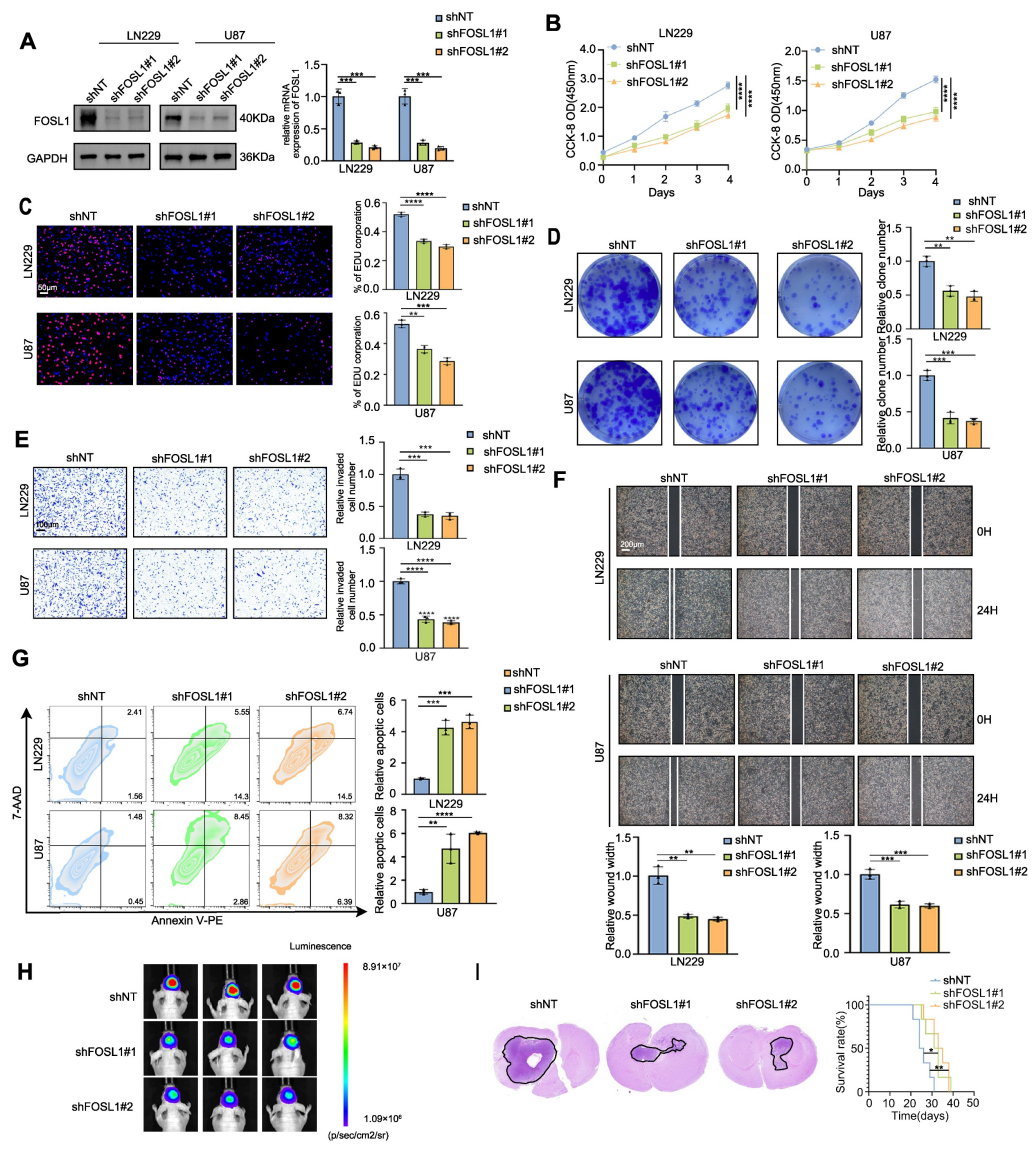
** Silencing FOSL1 attenuated the malignancies of GBM cells. A,** the knock-down efficiency of* shFOSL1* was measured by qRT-PCR (n = 3, with independent sample *t* test) and immunoblot analysis in LN229 and U87. **B-D,** the effect of FOSL1 knock-down on cell proliferation was evaluated by CCK-8 assays (n = 3, with one-way ANOVA test, **B**), EDU assays (n = 3, with independent sample *t* test, Scale bars, 50 μm, **C**), colony formation assays (n = 3, with independent sample *t* test, **D**). **E**, Cell matrigel invasion assays were performed to evaluate cell invasion in GBM cells following FOSL1 knock-down (n = 3, with independent sample *t* test). Scale bars, 100 μm. **F,** Wound-healing assays were performed to assess cell migration in GBM cells following FOSL1 knock-down (n = 3, with independent sample *t* test). Scale bars, 200 μm. **G,** Flow cytometry-based apoptosis analysis was used to evaluate cell apoptosis in GBM cells following FOSL1 knock-down (with independent sample* t* test).** H and I**, Representative bioluminescent images (**H**), H&E staining and Kaplan-Meier analysis (**I**) (n = 6 in each group, with log-rank test) of U87 orthotopic xenograft nude mice following FOSL1 knock-down. GAPDH was used as the loading control for normalization. *P < 0.05, **P < 0.01, ***P < 0.001, ****P < 0.0001. Data shown as mean ± SD. The immunoblotting experiments were repeated three times with similar results.

To elucidate the functional contribution of FOSL1 to GMB malignancy, exogenous silencing of *FOSL1* was performed in LN229 and U87 GBM cell cultures using lentiviral vectors expressing 2 distinct *FOSL1*-targeting shRNAs. Quantitative reverse transcription polymerase chain reaction (qRT-PCR) and immunoblot analyses confirmed a significant reduction of FOSL1 expression in the knockdown cells (Figure 1A). Subsequent *in vitro* cell proliferation assays, including Cell Counting Kit-8 (CCK-80, 5-ethynyl-2′-deoxyuridine (EdU) incorporation, and colony formation, indicated that suppression of FOSL1 significantly diminished proliferation in both LN229 and U87 cells (Figure 1B-D). Consistent with these findings, wound-healing and Matrigel-based invasion assays showed substantially reduced migratory and invasive capabilities following FOSL1 depletion (Figure 1E-F). In addition, flow cytometry-based apoptotic profiling revealed a marked increase in programmed cell death in FOSL1-silenced cells (Figure 1G). U87 cells, due to their high knockdown efficiency and consistent phenotypic response, were subsequently selected for establishing an orthotopic xenograft model in nude mice. *In vivo* experiments demonstrated that FOSL1 suppression significantly impaired GBM tumorigenicity, with Kaplan-Meier analysis confirming a notable extension of survival in xenografted mice with FOSL1 knockdown (Figure 1H-I).

To substantiate the oncogenic role of FOSL1, overexpression models were constructed in U373 and U251 cell lines based on their relatively low endogenous FOSL1 expression (Figure S4A). Forced elevation of FOSL1 expression significantly enhanced cell proliferation, migration, and invasion (Figure S4B-F), while concurrently reducing apoptotic cell death (Figure S4G). Consistent enhancement of tumorigenic capacity *in vivo* was also observed in FOSL1-overexpressing cells compared to controls (Figure S4H-I). Taken together, these experiments establish FOSL1 as a critical determinant of multiple malignant properties, including proliferation, migration, invasion, survival, and tumorigenesis, in GBM cells across both *in vitro* and *in vivo* contexts.

### FOSL1 promotes GBM malignancy through activation of NF-κB signaling

To investigate downstream mechanisms associated with FOSL1, gene set enrichment analysis (GSEA) was conducted based on differential gene expression across 2 stratification strategies: 1) subgroups defined by NMF clustering analysis (Figure S1D-E) and 2) samples stratified by *FOSL1* expression level (high versus low). As shown in Figure S5A-B, multiple oncogenic signaling pathways were enriched in FOSL1-associated transcriptomic profiles. To determine the most relevant pathways linked to elevated *FOSL1* expression in GBM, the top five enriched pathways (|NES| > 2.5, FDR < 0.05) were intersected across three independent analyses.

This comparison revealed consistent enrichment of oncogenic pathways including EMT, inflammatory response, and NF-κB signaling. Notably, NF-κB signaling emerged as the most represented pathway, being simultaneously enriched in the TCGA *FOSL1*-high subgroup (NES = 3.19), TCGA poor-prognosis cluster (NES = 3.38), and CGGA poor-prognosis cluster (NES = 3.27), all with a false discovery rate (FDR) < 0.001. Transcriptomic profiling following *FOSL1* knockdown further validated this association, as the TNFα-NF-κB signaling signature exhibited significant negative enrichment upon *FOSL1* depletion (Figure S5C). Consistent with this finding, expression levels of NF-κB downstream targets, including CXCL2, PTX3, and SERPINB2, were markedly reduced following *FOSL1* suppression (Figure S5D). These findings establish *FOSL1* as a positive regulator of NF-κB transcriptional activity in glioblastoma.

To functionally validate the FOSL1-dependent regulation of NF-κB signaling, LN229 and U87 cells were transduced with *shNT* or *shFOSL1* lentiviral constructs followed by TNFα stimulation. Comprehensive functional assays (CCK-8, EdU incorporation, colony formation, Matrigel invasion, and wound healing) demonstrated that FOSL1 knockdown substantially impaired cell proliferation, clonogenicity, migration, and invasion, with TNFα exposure partially restoring these malignant phenotypes (Figure S6A-D, Figure S7A, B). Flow cytometry analysis further confirmed the ability of TNFα to attenuate apoptosis induced by FOSL1 silencing (Figure S6E).

At the mechanistic level, immunoblotting indicated that FOSL1 depletion significantly attenuated IKKα phosphorylation and impeded IκBα degradation, thereby suppressing NF-κB pathway activation. TNFα treatment partially reversed these effects. In addition, *FOSL1* knockdown reduced total IKKα protein levels, supporting a role for FOSL1 in regulating IKKα expression, potentially at the transcriptional level, thereby modulating NF-κB signaling (Figure S6F).

**Figure 2:**
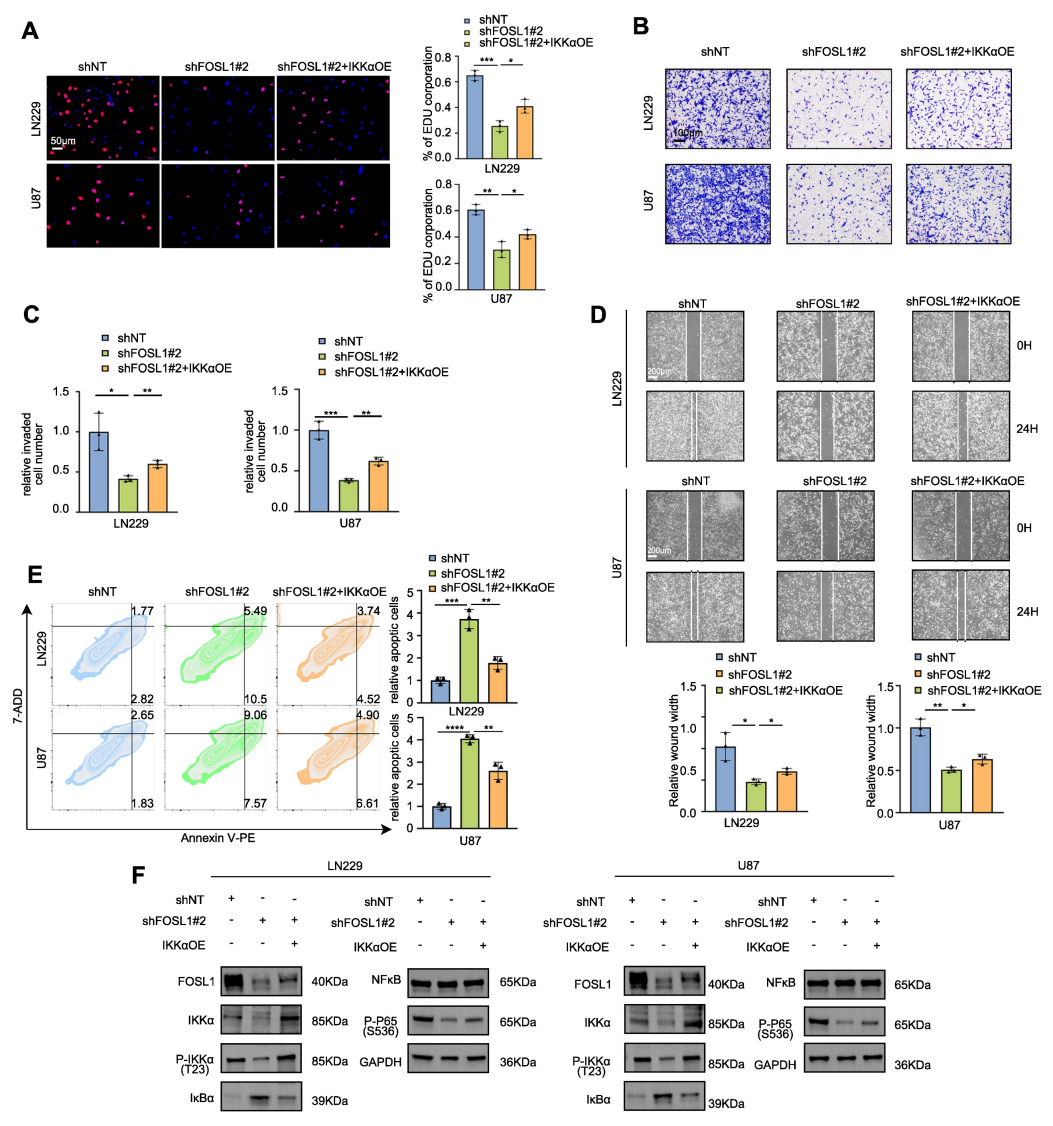
** FOSL1 promoted malignancies of GBM through activation of NF-κB signaling pathway. A,** EDU assay was employed to evaluate cell proliferation in LN229 and U87 cells following FOSL1 knock-down, with or without IKKα overexpression (n = 3, with independent sample *t* test). Scale bars, 50 μm. **B and C**, Cell matrigel invasion assays were performed to evaluate cell invasion in LN229 and U87 cells following FOSL1 knock-down, with or without IKKα overexpression (n = 3, with independent sample *t* test). Scale bars, 100 μm. **D,** Wound-healing assay was conducted to assess cell migration in LN229 and U87 cells following FOSL1 knock-down, with or without IKKα overexpression (n = 3, analyzed by independent sample *t* test). Scale bars, 200 μm. **E,** Flow cytometry-based apoptosis analysis was used to evaluate cell apoptosis in LN229 and U87 cells following FOSL1 knock-down, with or without IKKα overexpression (n = 3, with independent sample *t* test). **F**, immunoblot analysis was used to detect the expression of NF-κB related biomarkers in LN229 and U87 cells following FOSL1 knock-down, with or without IKKα overexpression. GAPDH was used as the loading control for normalization. *P < 0.05, **P < 0.01, ***P < 0.001, ****P < 0.0001. Data shown as mean ± SD. The immunoblotting experiments were repeated three times with similar results.

Furthermore, TNFα stimulation partially restored FOSL1 protein expression following lentiviral-mediated suppression. To directly examine the functional relationship between FOSL1 and IKKα, *IKKα* was reintroduced into FOSL1-silenced cells via overexpression constructs. IKKα restoration partially rescued the impaired malignant behaviors induced by FOSL1 depletion (Figure 2A-E, Figure S7C-D), and re-established NF-κB signaling activation by reinstating IKKα degradation (Figure 2F).

To further validate the role of FOSL1 in activating NF-κB signaling in GBM cells, confocal microscopy and nuclear-cytoplasmic fractionation assays were employed. The results demonstrated that elevated FOSL1 expression facilitated nuclear translocation of p65 (Figure S8A-B). Consistent with this observation, FOSL1 overexpression significantly increased RNA expression levels of downstream NF-κB target genes (Figure S8C). These findings support a mechanism in which FOSL1 promotes NF-κB activation by enhancing p65 nuclear import.

Collectively, these results demonstrate that FOSL1 promotes GBM malignancy *via* activation of TNFα-dependent NF-κB signaling.

### FOSL1 transcriptionally upregulates *IKKα* expression in GBM

**Figure 3:**
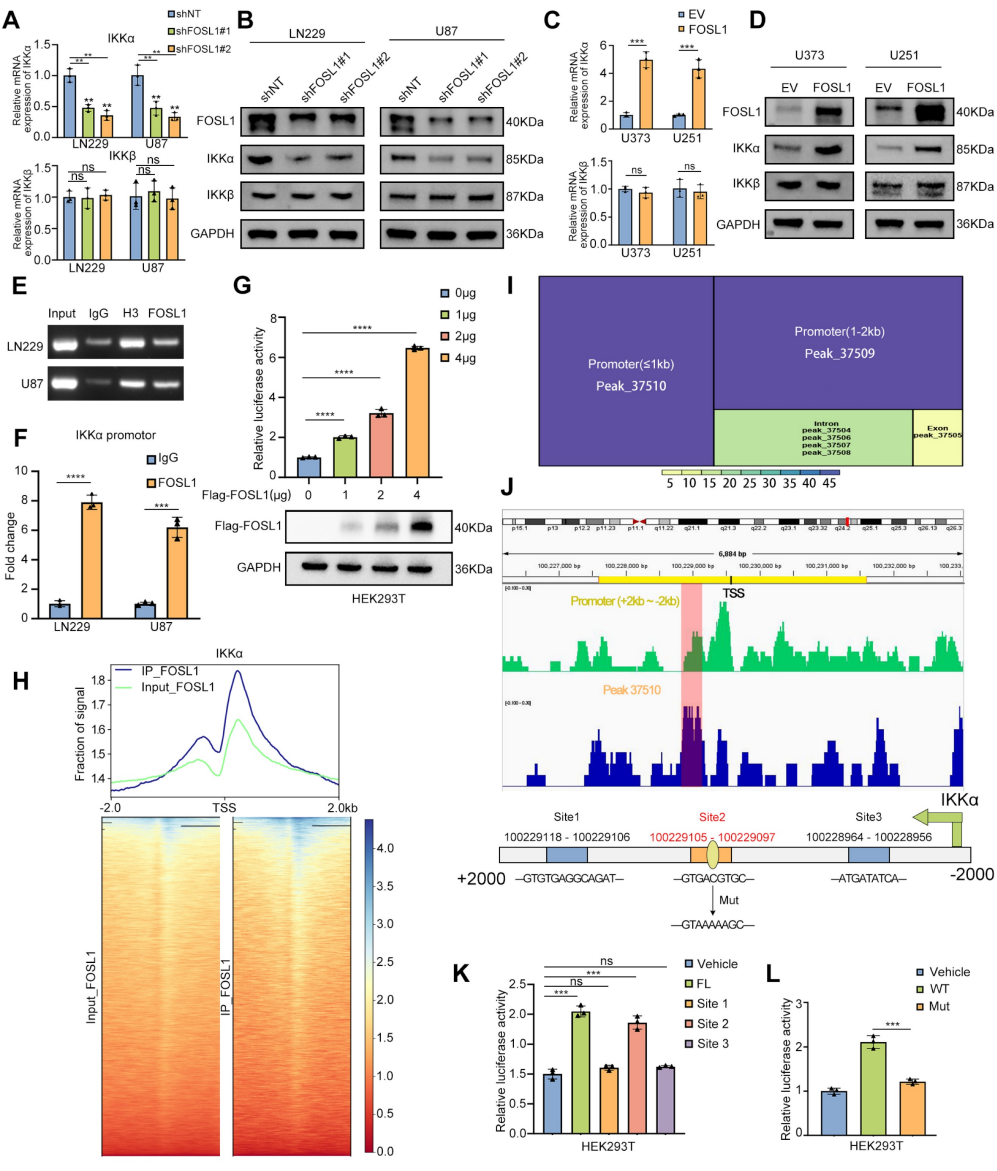
** FOSL1 transcriptionally up-regulated *IKKα* expression in GBM. A and B,** the qRT-PCR (n = 3, analyzed by independent sample *t* test) and immunoblot analysis were used to detect the expression of *IKKα* and *IKKβ* in LN229 and U87 cells following FOSL1 knock-down. **C and D,** the expression of *IKKα* and *IKKβ* were confirmed by qPCR (n = 3, with independent sample *t* test) and immunoblot analysis in U373 and U251 following FOSL1 overexpression. **E and F,** ChIP PCR and qRT-PCR (n = 3, analyzed by independent sample *t* test) analysis of FOSL1 binding to the *IKKα* promoter in LN229 and U87 cells. **G,** the direct activation in a concentration-dependent manner of *IKKα* by FOSL1 was validated by the luciferase activity assay in HEK293T cells (n = 3, analyzed by independent sample *t* test). **H,** Heatmaps of ChIP-seq signals (TSS ± 2 kb) for FOSL1 from the indicated groups.** I,** Motif analysis of *IKKα* promoter region FOSL1 binding sites. **J,** IGV visualization and schematic of the FOSL1 binding site on the *IKKα* promoter. **K,** HEK293T cells were transfected with 3 distinct deletion constructs of the *IKKα* promoter region, and luciferase activity was assessed using a luciferase activity assay. Statistical analysis revealed that the second binding site exhibited the most significant impact on promoter activity (n = 3, with independent sample *t* test).** L,** Luciferase activity was determined after mutation of the second FOSL1 site in the *IKKα* promoter in HEK293T cells. The luciferase activity of the wild-type (WT) *IKKα* promoter was significantly higher than that of mutant *IKKα* promoter (n = 3, with independent sample *t* test). GAPDH was used as the loading control for normalization. ns=not significant, *P < 0.05, **P < 0.01, ***P < 0.001, ****P < 0.0001. Data shown as mean ± SD. The immunoblotting experiments were repeated three times with similar results.

To evaluate the transcriptional regulation of *IKKα* by FOSL1, qRT-PCR and immunoblot analyses were performed to assess *IKKα* expression following FOSL1 knockdown or overexpression. FOSL1 silencing significantly reduced both mRNA and protein levels of *IKKα* in GBM cells (Figure 3A-B), whereas FOSL1 overexpression markedly elevated *IKKα* expression (Figure 3C-D). Notably, FOSL1 exerted minimal influence on *IKKβ* expression (Figure 3A-D). These findings support a working model in which FOSL1 activates NF-κB signaling by specifically upregulating *IKKα* at the transcriptional level rather than through post-translational modification.

To validate this mechanism, Chromatin Immunoprecipitation-PCR (ChIP-PCR) was performed and the result showed significant enrichment of FOSL1 at the *IKKα* promoter region (Figure 3E-F). Complementary dual-luciferase reporter assays in HEK293T cells indicated that FOSL1 increased *IKKα* promoter activity in a concentration-dependent manner (Figure 3G). ChIP-Seq analysis further confirmed a prominent FOSL1 binding peak across the *IKKα* promoter region (Figure 3H), and motif analysis identified peak 37510 as the strongest candidate for direct interaction (Figure 3I). Based on this peak, three potential FOSL1 binding sites were predicted using the JASPAR database (Figure 3J). Promoter deletion constructs covering these regions were generated, and dual-luciferase reporter assays in HEK293T cells identified the site spanning 100229105 - 100229097 as the primary binding locus for FOSL1 (Figure 3K). A point mutation introduced at this position resulted in a marked reduction in luciferase activity compared with the wild-type promoter, thereby confirming *IKKα* as a direct downstream target of FOSL1 and establishing a positive regulatory relationship between the two (Figure 3L). Collectively, these data demonstrate that FOSL1 drives transcriptional upregulation of *IKKα*, leading to activation of NF-κB signaling and promotion of malignant phenotypes in GBM cells.

To identify the specific JUN family member partnering with FOSL1 to form the AP-1 transcriptional complex, mass spectrometry (MS) analyses were performed and revealed interactions among c-JUN, JUNB, JUND, and FOSL1. DNA pull-down assays showed selective enrichment of JUNB at the *IKKα* promoter region, while both FOSL1 and JUNB failed to bind the mutant *IKKα* promoter construct (Figure S9A-B). Dual-luciferase reporter assays confirmed that JUNB overexpression enhanced transcriptional activation of the wild-type *IKKα* promoter but had no effect on the mutant variant (Figure S9C). ChIP assays further verified specific JUNB engagement with the *IKKα* promoter (Figure S9D-E). Confocal microscopy additionally revealed co-localization of FOSL1 and JUNB within the nuclei of GBM cells (Figure S9F), supporting their cooperative function within the AP-1 regulatory complex.

### IKKα phosphorates FOSL1 and enhances FOSL1 stability

As shown in Figure 2F and Figure S6F, TNFα stimulation and IKKα overexpression partially restored FOSL1 protein levels following lentiviral-mediated suppression, without altering *FOSL1* mRNA expression, implying a potential feedback mechanism linking FOSL1 and NF-κB signaling (Figure S10A). To further elucidate the FOSL1/IKKα/NF-κB regulatory axis, IKKα was either silenced or overexpressed in GBM cells. qRT-PCR and immunoblot analyses showed that IKKα knockdown significantly reduced FOSL1 protein levels without affecting FOSL1 transcript levels, whereas IKKα overexpression increased FOSL1 protein levels but not mRNA expression (Figure S10B-E). Additionally, ChIP assays were performed to evaluate potential regulation of FOSL1 by NF-κB transcriptional activity. The results showed that P65 exhibited no detectable effect on FOSL1 promoter binding or transcriptional activation (Figure S10F-G). Similarly, immunoblot analysis also demonstrated that *IKKβ* knockdown did not affect FOSL1 protein levels (Figure S10H). These findings suggest that IKKα modulates FOSL1 expression primarily through post-translational modification rather than transcriptional control.

**Figure 4:**
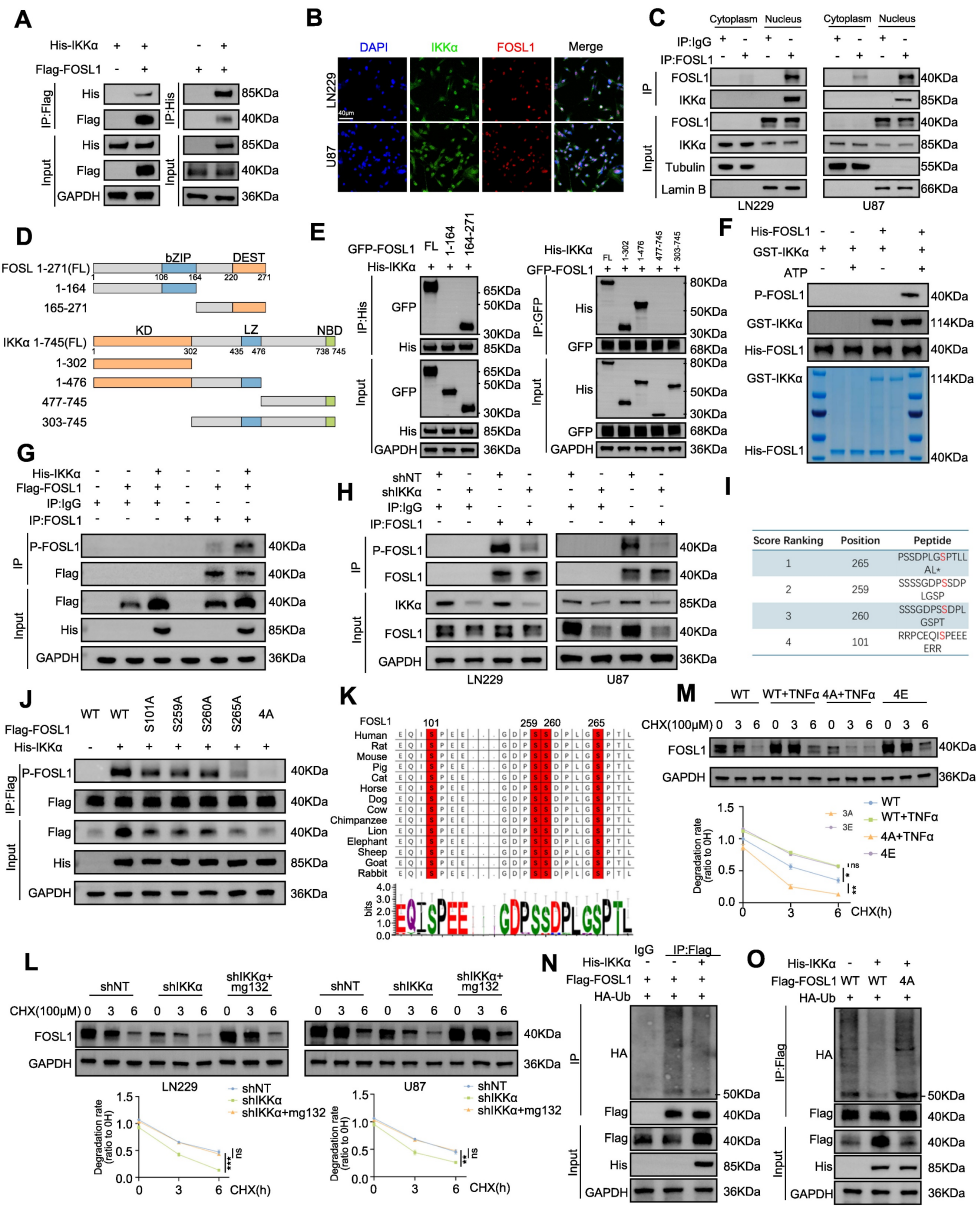
**IKKα phosphorated FOSL1 thus enhanced the stability of FOSL1. A**, immunoblot analysis of Flag-FOSL1 and His-IKKα expression in a co-IP assay performed using protein A/G magnetic beads and anti-Flag (left) or anti-His (right) primary antibody in HEK293T cells. **B,** Representative image of the co-localization of FOSL1 (red) and IKKα (green) protein in LN229 (upper) and U87 (bottom) cells observed by confocal microscope. Scale bars, 40 μm. **C,** Subcellular fractionation followed by co-IP assay was performed to investigate FOSL1-IKKα interactions in LN229 (left) and U87 (right) cells. Tubulin and Lamin B served as cytoplasmic and nuclear loading controls, respectively, for normalization. **D,** Schematic representation of full-length (FL) FOSL1 and IKKα, along with their different truncation mutants. bZIP: basic leucine zipper, c-DEST: C-terminal unstructurized destabilizing area; KD: Kinase domain, LZ: Leucine-zipper NBD: NEMO-binding domain. **E,** (left) GFP-FOSL1 FL or indicated truncation mutants were co-expressed with His-IKKα in HEK293T cells. (right) His-IKKα FL or indicated truncation mutants were co-expressed with GFP-FOSL1 in HEK293T cells. **F,** to investigate the kinase activity of IKKα toward FOSL1, *in vitro* kinase assays were performed using recombinant active IKKα and recombinant FOSL1 as substrate. Immunoblot analysis and coomassie brilliant blue staining were used to verify FOSL1 phosphorylation and equal protein loading, respectively. **G,** Co-IP assays were performed in HEK293T cells transfected with or without Flag-FOSL1 and His-IKKα plasmid. **H,** Co-IP assays were performed in LN229(left) and U87(right) cells with or without FOSL1 knock-down. **I,** Phosphorylated residues in FOSL1 predicted by GPS 6.0. **J,** Co-IP assays were performed in HEK293T cells transfected with or without FOSL1 mutant plasmids (Mutation of the primary phosphorylation site to alanine). **K,** Sequence conservation analysis of relevant amino acids of FOSL1. **L,** LN229 and U87 cells with or without IKKα knock-down, followed by cycloheximide (CHX; 100 μM) for 0, 3, 6 h. Density of FOSL1 expression was quantified by ImageJ. **M,** HEK293T cells were transfected as indicated followed by cycloheximide (CHX; 100 μM) for 0, 3, 6 h. Density of FOSL1 expression was quantified by ImageJ. **N,** HEK293T cells were transfected as indicated followed by immunoprecipitation with anti-Flag primary antibody. **O,** HEK293T cells were transfected with or without FOSL1 mutant plasmids, followed by immunoprecipitation with anti-Flag primary antibody. GAPDH was used as the loading control for normalization. Data shown as mean ± SD. The immunoblotting experiments were repeated three times with similar results.

To further validate the physical interaction between FOSL1 and IKKα, exogenous Flag-FOSL1 and His-IKKα were co-expressed in HEK293T cells, followed by co-immunoprecipitation (co-IP). Reciprocal pulldown using anti-Flag or anti-His antibodies confirmed the association between His-IKKα and Flag-FOSL1 (Figure 4A). Consistently, endogenous interaction between native FOSL1 and IKKα in GBM cell lines was confirmed by co-IP assays (Figure S10I). GST pull-down assays using purified GST-FOSL1 and His-IKKα further confirmed a direct binding interaction, supporting the formation of a FOSL1-IKKα complex (Figure S10J). Confocal microscopy showed nuclear co-localization of FOSL1 and IKKα (Figure 4B), and nuclear-cytoplasmic fractionation combined with co-IP identified the nucleus as the principal interaction site (Figure 4C). Taken together, these results collectively establish a direct physical interaction between FOSL1 and IKKα.

To define the domains responsible for this interaction, GFP-FOSL1 and His-IKKα truncation mutants with deletions in different domains were constructed for co-IP analysis. These assays identified the c-DEST domain of FOSL1 (residues 164-271) and the N-terminal kinase domain of IKKα (residues 1-302) as essential for their interaction (Figure 4D-E). In summary, these findings demonstrate that FOSL1 and IKKα directly interact through the c-DEST domain of FOSL1 and the kinase domain of IKKα, providing a mechanistic basis for their cooperative function within the FOSL1/IKKα signaling axis.

To determine whether FOSL1 functions as a substrate for IKKα-mediated phosphorylation, immunoblot analysis was performed and showed a positive correlation between phosphorylated FOSL1 (p-FOSL1) and IKKα expression levels (Figure S10K-L). *In vitro* kinase assays further confirmed direct phosphorylation of purified FOSL1 by IKKα (Figure 4F). In HEK293T cells overexpressing Flag-FOSL1, co-IP assays demonstrated elevated levels of phosphorylated Flag-FOSL1 (Figure 4G), while endogenous co-IP in GBM cells revealed that IKKα knockdown reduced FOSL1 phosphorylation (Figure 4H). To exclude phosphorylation effects mediated by ERK2, a known regulator of FOSL1, IKKα-overexpressing cells were treated with the ERK inhibitor ulixertinib. The inhibitor only partially attenuated the IKKα-induced increase in p-FOSL1 levels, suggesting that IKKα-dependent phosphorylation of FOSL1 constitutes a distinct post-transcriptional regulatory mechanism (Figure S10M).

Phosphosite prediction analysis (GPS 6.0, https://gps.biocuckoo.cn/) 34 identified four conserved serine residues (Ser101, Ser259, Ser260, and Ser265) as putative IKKα phosphorylation targets (Figure 4I). Mutational screening revealed that substitution at FOSL1^Ser265^ significantly reduced FOSL1 phosphorylation, suggesting that Ser265 represents the dominant IKKα-mediated phosphorylation site (Figure 4J). Additionally, sequence alignment confirmed that Ser265 is evolutionarily conserved across multiple species (Figure 4K), supporting its biological relevance in FOSL1 regulatory function.

To further assess the impact of IKKα-mediated phosphorylation on FOSL1 stability, cycloheximide was applied to inhibit protein synthesis, revealing that IKKα knockdown shortened the half-life of FOSL1, whereas proteasome inhibition with MG132 restored FOSL1 stability (Figure 4L). To directly establish phosphorylation-dependent regulation, two genetic variants of FOSL1 were employed: a phospho-deficient mutant (4A-FOSL1; Ser-to-Ala) and a phosphomimetic mutant (4E-FOSL1; Ser-to-Glu) targeting the four serine residues identified. In HEK293T cells expressing these constructs, TNFα increased the stability of WT-FOSL1 but not the non-phosphorylatable 4A-FOSL1, while the phosphomimetic 4E-FOSL1 mutant exhibited greater stability than WT-FOSL1(Figure 4M). In parallel, IKKα knockdown increased polyubiquitination of endogenous FOSL1 in GBM cells (Figure S10N), whereas IKKα overexpression reduced FOSL1 ubiquitination in HEK293T cells—a regulatory effect that was abolished in the 4A-FOSL1 mutant (Figure 4N-O). Collectively, these findings indicated that IKKα-mediated phosphorylation stabilizes FOSL1 by inhibiting its ubiquitination and subsequent proteasomal degradation.

### UCHL3 is essential for IKKα-mediated stabilization of FOSL1

**Figure 5:**
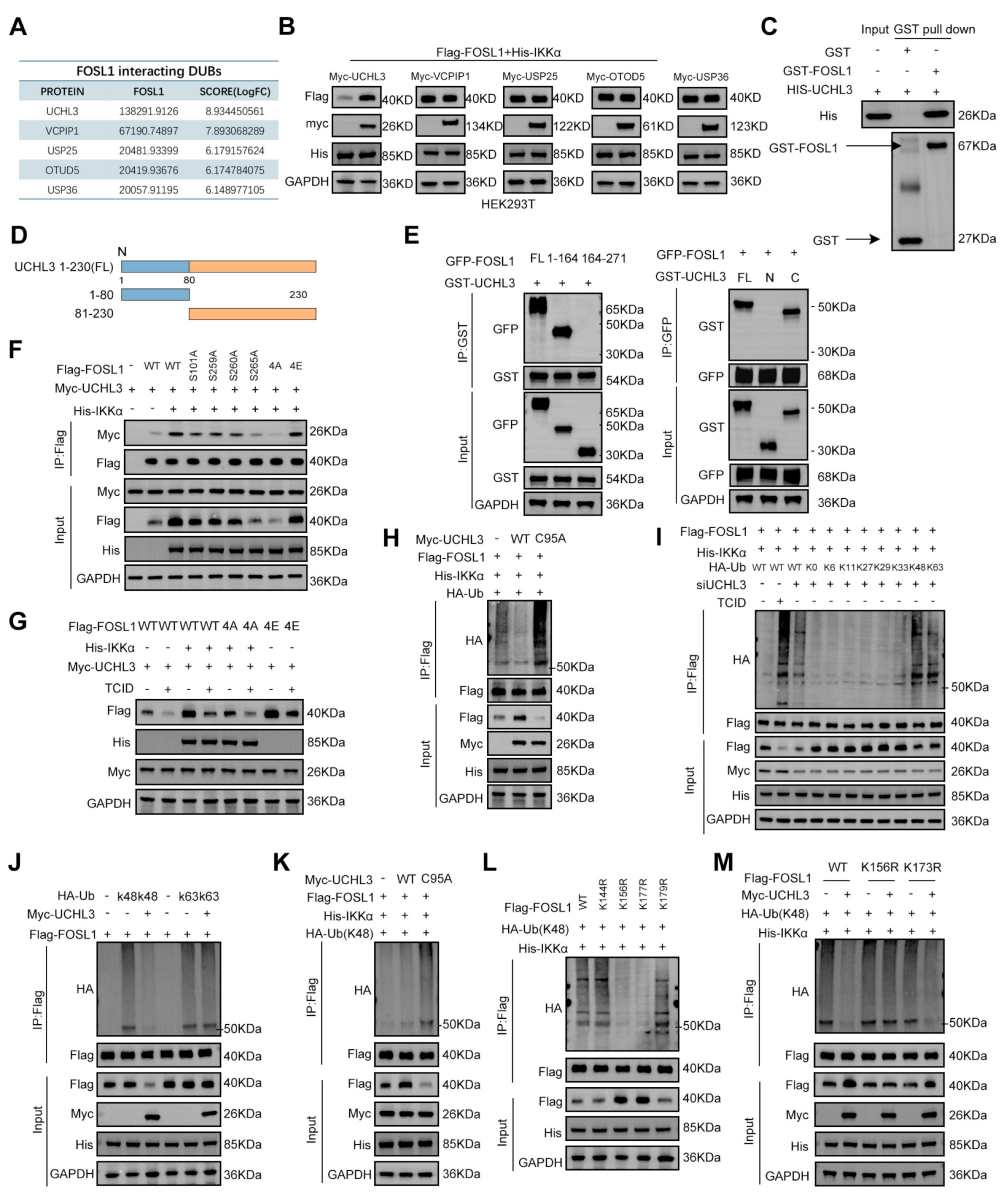
**UCHL3 was essential for IKKα-mediated stabilization of FOSL1. A,** MS analysis identified potential deubiquitinating enzymes (DUBs) interacting with FOSL1. **B,** FOSL1 protein levels were assessed in HEK293T cells overexpressing myc-tagged DUB candidates (UCHL3, VCPIP1, USP25, OTUD5, or USP36) alongside Flag-FOSL1. **C,** the physical interaction between FOSL1 and UCHL3 was confirmed by GST pull-down assays. GST protein alone served as the negative control. **D,** Schematic representation of UCHL3 FL and its truncation mutants. **E,** (left) GFP-FOSL1 FL or indicated truncation mutants were co-expressed with GST-UCHL3 in HEK293T cells. (right) GST-UCHL3 FL or indicated truncation mutants were co-expressed with GFP-FOSL1 in HEK293T cells. **F,** IKKα overexpression promotes FOSL1-UCHL3 interaction. Lysates of HEK293T cells transfected as indicated were immunoprecipitated with anti-Flag primary antibody, followed by immunoblot analysis. **G,** HEK293T cells were transfected as indicated with or without UCHL3 inhibitors treatment. Lysates were used for immunoblot analysis to measure the protein levels of FOSL1. **H,** UCHL3 decreases ubiquitination of FOSL1. HEK293T cells transfected as indicated immunoprecipitated with anti-Flag primary antibody, followed by immunoblot analysis. **I,** HEK293T cells were transfected with Flag-FOSL1, various HA-ubiquitin mutants and *siUCHL3*, followed by treatment with or without UCHL3 inhibitors TCID (10 μM, 24 h). **J,** HEK293T cells were co-transfected with Flag-FOSL1, various HA-ubiquitin mutants (K48 and K63). **K,** Co-IP assays were performed in HEK293T cells transfected with or without catalytically inactive UCHL3 mutant (C95A) plasmids and HA-UB(K48). **L,** HEK293T cells were co-transfected with various Flag-FOSL1 mutants. **M,** Co-IP assays were performed in HEK293T cells transfected with or without FOSL1 mutant plasmids (K156R and K173R) and HA-UB (K48). GAPDH was used as the loading control for normalization. The immunoblotting experiments were repeated three times with similar results.

To elucidate how IKKα-dependent phosphorylation reduces FOSL1 ubiquitination, co-IP assays using anti-FOSL1 antibodies in U87 cell lysates followed by MS identified five candidate deubiquitinating enzymes (DUBs 35) interacting with FOSL1: UCHL3, VCPIP1, USP25, OTUD5, and USP36 (Figure 5A). Overexpression of each candidate DUB in HEK293T cells revealed that only the overexpression of UCHL3 significantly increased FOSL1 protein levels, indicating that UCHL3 is the primary DUB responsible for FOSL1 stabilization (Figure 5B). Co-IP assays further validated the interaction between FOSL1 and UCHL3 in GBM cells (Figure S11A-B), and GST-pull-down assays using purified GST-FOSL1 and His-UCHL3 proteins substantiated their direct binding (Figure 5C). Domain mapping using truncation constructs of both FOSL1 and UCHL3 alongside co-IP experiments demonstrated that the bZIP domain of FOSL1 and the C-terminal region of UCHL3 were required for their interaction (Figure 5D-E). Confocal microscopy revealed pronounced nuclear co-localization of FOSL1, IKKα, and UCHL3 in GBM cells (Figure S12A-B). Nuclear-cytoplasmic fractionation followed by co-IP further established that the FOSL1-IKKα-UCHL3 complex is formed specifically within the nuclear compartment (Figure S12C).

To further delineate the regulatory interactions among FOSL1, UCHL3, and IKKα, qRT-PCR and immunoblot analyses were performed. UCHL3 knockdown in GBM cells resulted in a decrease in FOSL1 protein levels, while leaving FOSL1 mRNA levels unchanged, and did not affect IKKα protein or transcript levels (Figure S11C-D). Treatment with TCID, a selective UCHL3 inhibitor, produced consistent results (Figure S11E). Similarly, IKKα knockdown failed to alter UCHL3 protein or mRNA expression (Figure S11F-G), indicating the absence of direct transcriptional regulation between IKKα and UCHL3. To determine whether IKKα-mediated phosphorylation modulates the interaction between FOSL1 and UCHL3, various FOSL1 mutants were expressed in HEK293T cells. IKKα enhanced the binding between FOSL1 and UCHL3, whereas phosphorylation-deficient mutants, particularly 4A-FOSL1, showed weakened association with UCHL3 (Figure 5F). Notably, IKKα overexpression elevated FOSL1 protein levels in a manner that was abolished by UCHL3 inhibition, while the phospho-mimetic 4E-FOSL1 mutant retained enhanced stability (Figure 5G). Endogenous IKKα overexpression also prolonged the FOSL1 half-life, whereas UCHL3 inhibition accelerated its turnover (Figure S11H). Together, these findings suggest that IKKα-dependent phosphorylation facilitates the interaction between FOSL1 and UCHL3, thereby promoting FOSL1 stabilization.

Conversely, UCHL3 knockdown reduced FOSL1 protein abundance, which was reversed by t the proteasome inhibitor MG132 (Figure S11I). Expression of the catalytically inactive UCHL3 mutant (C95A) in HEK293T cells demonstrated that stabilization of FOSL1 requires UCHL3 enzymatic activity (Figure 5H). Furthermore, ectopic UCHL3 expression rescued TCID-induced FOSL1 degradation in GBM cells (Figure S11J). These results indicate that UCHL3-mediated FOSL1 stabilization is critically dependent on deubiquitinase activity and operates through the ubiquitin-proteasome system.

To determine the specificity of UCHL3 enzymatic activity, ubiquitin mutants with defined linkage patterns were overexpressed in HEK293T cells. UCHL3 effectively cleaved both K48- and K63-linked polyubiquitin chains (Figure 5I). Given that K48-linked ubiquitination predominantly targets proteins for proteasomal degradation, whereas K63-linked chains contribute to non-proteasomal regulatory processes 36, the precise ubiquitin linkage responsible for regulating FOSL1 stability was subsequently evaluated. Overexpression of UCHL3 significantly reduced K48-linked ubiquitination of FOSL1, whereas the catalytically inactive UCHL3-C95A mutant failed to do so (Figure 5J-K). Predictive analysis using GSP-Uber (https://gpsuber.biocuckoo.cn/) 37 identified four candidate ubiquitination sites on FOSL1 (K144, K156, K173, and K179) (Figure S11K). Site-directed mutagenesis demonstrated that substitutions at K156R and K173R markedly reduced K48-linked ubiquitination of FOSL1 (Figure 5L). Notably, UCHL3 overexpression did not reverse the ubiquitination phenotype of the K156R mutant (Figure 5M), suggesting K156 as the key residue for UCHL3-mediated deubiquitination. Additionally, sequence alignment demonstrated that K156 is evolutionarily conserved across FOSL1 orthologs (Figure S11L). In summary, these findings establish that UCHL3 selectively removes K48-linked polyubiquitin chains from FOSL1 at K156, thereby stabilizing FOSL1 protein through deubiquitinase activity (Figure S12D).

### CUL3 acts as an E3 ubiquitin ligase for FOSL1

To identify the E3 ubiquitin ligase responsible for FOSL1 ubiquitination and degradation, MS analysis identified three candidate ligases: CUL3, TOM1, and AMFR. Co-IP assays revealed that CUL3 overexpression markedly reduced FOSL1 protein levels, implicating CUL3 as a likely E3 ligase for FOSL1 (Figure S13A). Consistent with this, CUL3 knockdown increased, while CUL3 overexpression decreased, FOSL1 protein levels without altering FOSL1 mRNA, as confirmed by immunoblotting and qRT-PCR (Figure S13B-E). Additional Co-IP assays in GBM cells confirmed a direct interaction between endogenous FOSL1 and CUL3 (Figure S13F).

To assess the effect of CUL3 on FOSL1 stability, protein synthesis inhibition with cycloheximide showed that CUL3 overexpression reduced the half-life of FOSL1, whereas IKKα co-expression restored FOSL1 stability (Figure S13G). Confocal microscopy confirmed nuclear co-localization of FOSL1, IKKα, and CUL3 (Figure S13H). However, IKKα knockdown did not affect CUL3 protein or mRNA levels, indicating no direct transcriptional relationship between IKKα and CUL3 (Figure S13I). Overexpression of FOSL1 mutants in HEK293T cells demonstrated that IKKα enhanced the interaction between FOSL1 and CUL3, whereas the phosphorylation-deficient 4A-FOSL1 mutant exhibited diminished CUL3 binding and reduced ubiquitination (Figure S13G-K).

Collectively, these results demonstrate that CUL3 functions as an E3 ubiquitin ligase for FOSL1, and that the strength of the CUL3-FOSL1 interaction is modulated by IKKα-mediated phosphorylation, thereby influencing the ubiquitination and stability of FOSL1.

### Development and evaluation of a nanocapsuled siRNA delivery system for GBM therapy

**Figure 6:**
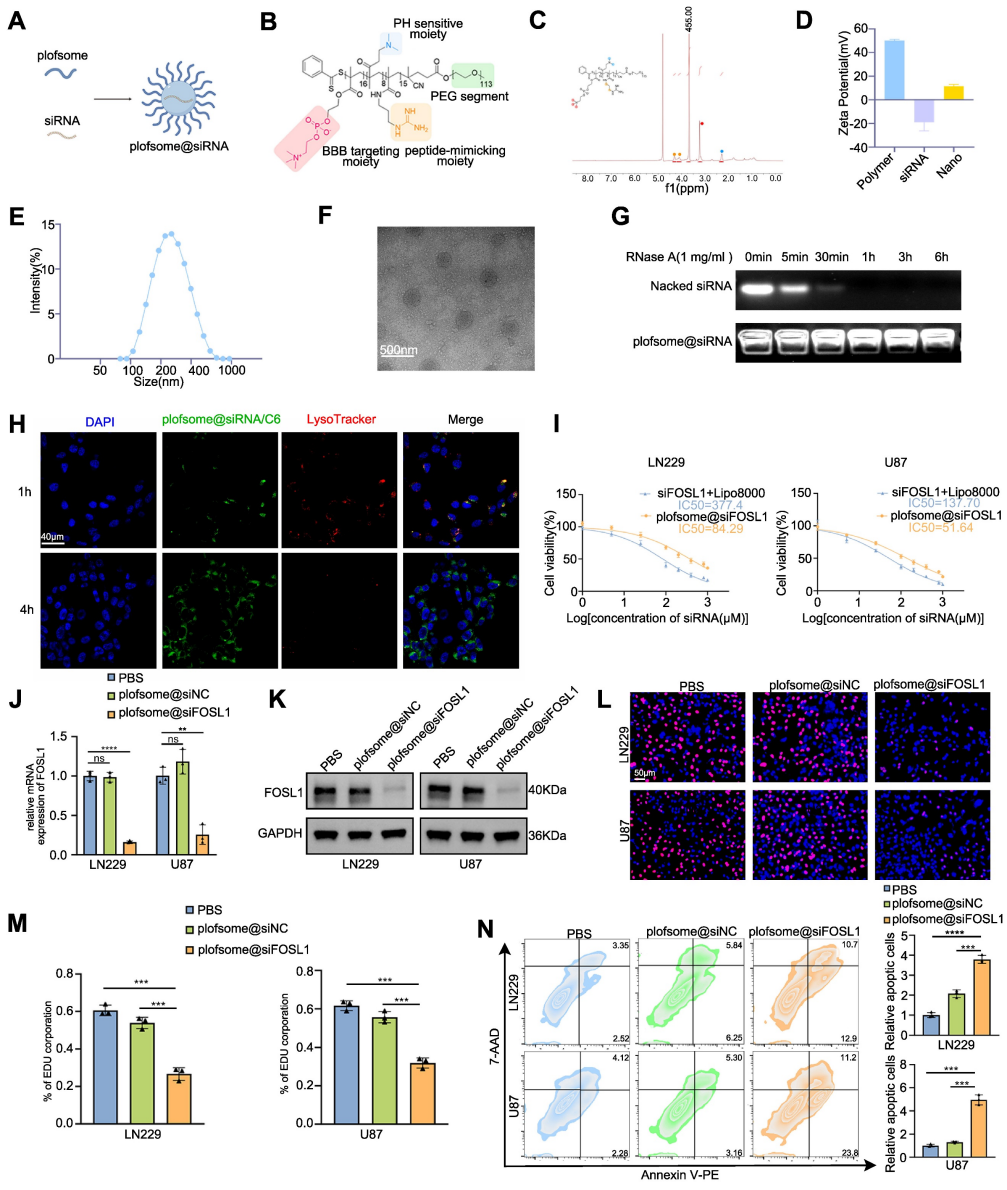
** Development and evaluation of a nanocapsuled siRNA delivery system for GBM therapy. A,** Illustration of the formation of plofsome@*siFOSL1*. **B,** Chemical structures of PPPM. **C,** ^1^H NMR spectrum of PPPM in D_2_O, 400 MHz. **D,** Hydrodynamic size distribution of plofsome@*siFOSL1* determined by DLS. **E,** Zeta potential of PPPM, siRNA and plofsome@*siFOSL1*.** F,** TEM images of plofsome@*siFOSL1*. Scale bars, 500 nm. **G,** Naked siRNA and plofsome@*siNC* were treated with RNase and integrity was analyzed by agarose gel electrophoresis to assess stability of siRNA. **H,** Endosomal escape of plofsome@*siNC*/C6 in U87 cells. Confocal microscope images show (from left to right): nuclei stained with DAPI (blue), C6-plofsome@*siNC* (green), endosomes stained with LysoTracker (red) and merged images. Scale bars, 40 μm. **I,** The viability of LN229 and U87 cells was measured by CCK-8 assay after incubation with different plofsomes.** J and K,** Knock-down efficiency of plofsome@*siFOSL1* was measured by qRT-PCR (n = 3, with independent sample *t* test) and immunoblot analysis in LN229 and U87. **L and M,** EDU assay was employed to evaluate cell proliferation in LN229 and U87 cells following treated by PBS, plofsome@*siNC* or plofsome@*siFOSL1* (n = 3, with independent sample *t* test). Scale bars, 50 μm. **N,** Flow cytometry-based apoptosis analysis was used to assess cell apoptosis in LN229 and U87 cells following treated by PBS, plofsome@*siNC* or plofsome@*siFOSL1* (n = 3, with independent sample *t* test). GAPDH was used as the loading control for normalization. **P < 0.01, ***P < 0.001, ****P < 0.0001. Data shown as mean ± SD. The immunoblotting experiments were repeated three times with similar results.

The above findings identify *FOSL1* as a compelling therapeutic target in GBM, where selective suppression of its expression may inhibit tumor progression. However, direct pharmacological targeting of *FOSL1* remains challenging due to the lack of intrinsic enzymatic activity typical of TFs 38-40. Recent advances in nanomedicine have demonstrated the utility of nanoplatform-assisted siRNA delivery for targeted gene silencing in GBM, providing a viable strategy for precision intervention 41-43. Based on this rationale, a nano-based siRNA delivery system (plofsome@*siFOSL1*) was developed using electrostatic assembly between positively charged PPPM block copolymer and negatively charged siRNA (Figure 6A-B). PPPM was synthesized using a reversible addition-fragmentation chain transfer (RAFT) polymerization method established in previous work 44, 45, followed by polymerization with 2-(Methacryloyloxy) ethyl 2-(Trimethylammonio) ethyl Phosphate (MPC), N-(3-Aminopropyl) methacrylamide (APMA), and 2-(Dimethylamino) ethyl methacrylate (DMAEMA). Subsequent guanidinylation of the amino groups increased the overall positive charge of PPPM, enabling spontaneous siRNA encapsulation through electrostatic condensation. The incorporation of MPC units facilitates blood-brain barrier (BBB) permeation by serving as substrates for nicotinic acetylcholine receptors (nAChRs) and choline transporters (ChTs), which are highly expressed in endothelial cells 46, 47. APMA contributes to pH responsiveness, ensuring efficient lysosomal escape and cytosolic release of siRNA upon cellular uptake. Successful PPPM synthesis was verified by ^1^H NMR spectroscopy, which revealed the presence of approximately 16 MPC, 8 DMAEMA, and 15 guanidine moieties per polymer chain based on characteristic signal peaks (Figure 6C). Guanidine-mediated condensation yielded a final siRNA loading of 6.2% in plofsome@*siFOSL1*. The shift in zeta potential from negatively charged naked siRNA to positively charged plofsome@*siFOSL1* further confirmed successful complex formation (Figure 6D). Dynamic light scattering (DLS) and transmission electron microscopy (TEM) images revealed a hydrodynamic diameter of approximately 250 nm and a spherical morphology in aqueous solution (Figure 6E-F). Agarose gel retardation assays showed that plofsome@*siFOSL1* protected siRNA from ribonuclease-mediated degradation for at least 6 h, confirming its stability, whereas naked *siFOSL1* was fully degraded within 30 min (Figure 6G). Additionally, plofsome@*siFOSL1* retained colloidal integrity and siRNA protection capability in 50% FBS for up to 24 h, demonstrating satisfactory serum stability despite a particle size of ~250 nm and a positive zeta potential (Figure S14A).

The cellular uptake and intracellular release behavior of plofsome@*siFOSL1* were examined using CLSM. Coumarin-6 (C6)-labeled plofsome@*siFOSL1* exhibited strong co-localization with lysosomes at 1 h post-incubation. By 4 h, a diffuse cytoplasmic distribution of green fluorescence was observed alongside attenuation of LysoTracker signal, a pattern indicative of efficient lysosomal escape and cytosolic release of the siRNA cargo (Figure 6H). Flow cytometry analysis produced results consistent with the CLSM-based observations (Figure S14B). The therapeutic efficacy of plofsome@*siFOSL1* in mediating *FOSL1* gene silencing and suppression of malignant phenotypes in GBM cells was subsequently evaluated. CCK-8 assays revealed that plofsome@*siFOSL1* exhibited the highest cytotoxicity potency, with an IC50 of 51.64 μM in U87 cells, significantly lower than that of naked *siFOSL1* delivered by transfection (IC50 = 137.7 μM). Corresponding IC50 values in LN229 cells were 84.29 μM for plofsome@*siFOSL1* and 377.4 μM *siFOSL1*, confirming superior knockdown efficiency and antitumor activity of the nanoparticle formulation (Figure 6I). The IC50 of plofsome@*siFOSL1* was subsequently applied for all downstream cellular assays. qRT-PCR and immunoblot analysis performed 48 h after treatment confirmed significant reductions in both mRNA and protein levels of *FOSL1* relative to the PBS and plofsome@siNC controls (Figure 6J-K). EdU proliferation assays further demonstrated a significant reduction in cell proliferation following plofsome@*siFOSL1* treatment (Figure 6L-M). Consistently, flow cytometry-based apoptosis analysis revealed a significant increase in apoptotic cell death, with apoptosis rates rising from 12.09% to 23.6% in LN229 cells and from 8.46% to 35% in U87 cells, relative to plofsome@*siNC* (Figure 6N). In summary, these findings demonstrate that plofsome@*siFOSL1* achieves efficient intracellular delivery of siRNA, robust FOSL1 silencing, and significant antitumor efficacy in GBM cells *in vitro*.

### *In vivo* evaluation of plofsome@*siFOSL1* for GBM therapy

The BBB permeation ability of plofsome@*siFOSL1* was first evaluated using an *in vitro* BBB model consisting of HCMEC/D3 cells in the upper chamber and U87 cells in the lower chamber of a transwell system. Confocal imaging revealed progressive accumulation of green fluorescence in U87 cells at both 1 and 4 h, showing time-dependent transendothelial transport and BBB permeation (Figure S15A-B). Subsequently, the *in vivo* pharmacokinetic profile of plofsome@*siFOSL1* was evaluated *via* intravenous injection into nude mice bearing U87-luc orthotopic xenografts. Near-infrared fluorescence imaging at 1, 3, 6, 12, 24, and 48 h post-injection revealed detectable intracranial accumulation as early as 1 h, with maximal brain-associated fluorescence observed at 24 h, followed by gradual signal decline by 48 h (Figure S15C-D). *Ex vivo* fluorescence imaging of harvested organs at 24 h demonstrated that approximately 12.43% of total fluorescence intensity localized within the brain, underscoring the effective targeting capability of plofsome-based siRNA delivery (Figure S15E). To characterize the circulation dynamics of the nanocapsule formulation, plofsome@*siNC*/IR780 was intravenously administered to tumor-free mice. Quantitative measurement of IR780-siRNA levels in plasma samples revealed a systemic half-life of approximately 30 min (Figure S15F), indicating an adequate retention window for effective tumor site accumulation.

**Figure 7:**
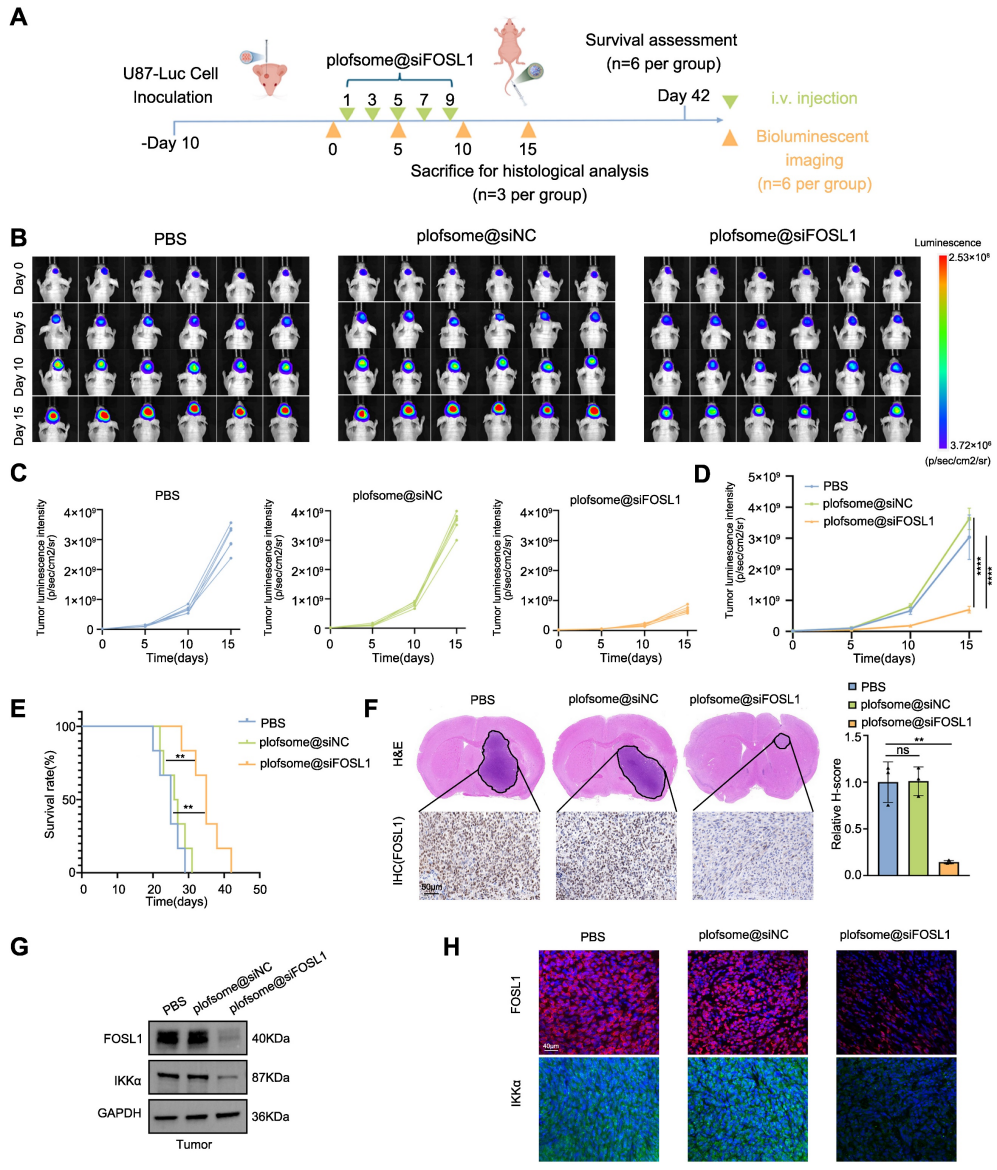
***In Vivo* evaluation of plofsome@*siFOSL1* for GBM therapy. A,** Schematic illustration of the* in vivo* therapy timeline design. **B,** *In vivo* bioluminescence imaging of U87MG-Luc tumor growth with different treatments. **C and D,** Individual tumor growth curves (**C**) and average tumor growth kinetics (**D**) by analyzing the normalized intensities of the bioluminescence signals (n = 3, with one-way ANOVA test).** E,** Kaplan-Meier curves show survival rates of U87MG-Luc tumor-bearing mice in different treatment groups (n = 6 in each group, with log-rank test). **F,** H&E staining of whole brain sections from treated mice (top). IHC staining of FOSL1 expression in brain sections following different treatments (bottom). Quantitative analysis using the H-score system was performed for IHC evaluation. Scale bars, 50 μm. **G and H,** Immunoblot and immunofluorescence analysis of FOSL1 protein levels in tumor tissues after plofsome@*siFOSL1* treatment. GAPDH was used as the loading control for normalization. Scale bars, 40 μm. **P < 0.01, ****P < 0.0001. Data shown as mean ± SD. The immunoblotting experiments were repeated three times with similar results.

To further assess the therapeutic efficacy of plofsome@*siFOSL1* against GBM, a U87-Luc orthotopic xenograft model was established using stereotactic injection of U87-Luc cells. Tumor-bearing nude mice received intravenous administration of plofsome@*siFOSL1* on days 1, 3, 5, 7, and 9, and tumor progression was monitored using *in vivo* fluorescence imaging on days 5, 10, and 15 (Figure 7A). Rapid tumor expansion was observed in the PBS and plofsome@*siNC* groups, whereas plofsome@*siFOSL1* treatment resulted in a relative reduction of luciferase bioluminescence, with the weakest bioluminescence intensity observed at day 15, indicating effective tumor growth suppression (Figure 7B). Statistical analysis showed that tumor bioluminescence increased by 122.35-fold in the PBS group and 156.14-fold in the plofsome@*siNC* group over the 15-day period, whereas the plofsome@*siFOSL1* group showed only a 36.19-fold increase. Compared to the PBS group, plofsome@*siFOSL1* achieved a tumor inhibition rate of 85.88% (Figure 7C-D). Kaplan-Meier survival analysis revealed significantly prolonged overall survival following plofsome@*siFOSL1* treatment, with a median survival duration of 35 days compared to 25 days and 26.5 days for PBS and plofsome@*siNC*, respectively (Figure 7E).

At the conclusion of the treatment regimen, brain tissues were collected on day 15 for histopathological examination. Hematoxylin-eosin (H&E) staining revealed substantially reduced tumor mass in the plofsome@*siFOSL1*-treated group relative to controls. IHC staining further revealed a marked reduction in *FOSL1* expression within tumor regions following plofsome@*siFOSL1* administration (Figure 7F). To evaluate *in vivo* molecular target engagement, immunoblotting and immunofluorescence analysis of tumor tissue confirmed that plofsome@*siFOSL1* treatment significantly reduced the expression of both FOSL1 and IKKα (Figure 7G-H).

To further evaluate the biosafety of plofsome@*siFOSL1*, histopathological examination and blood biochemical testing were conducted. No detectable tissue damage or structural abnormalities were observed in major organs—including heart, liver, spleen, lung, kidney, and non-tumor brain—after 15 days of treatment (Figure S16A). Furthermore, serum biochemical parameters revealed no impairment of hepatic or renal function in plofsome@*siFOSL1*-treated mice (Figure S16B). Assessment of FOSL1 expression across multiple tissues demonstrated a significant decrease exclusively within tumor tissue, with no corresponding decrease in liver, kidney, or lung samples, supporting the tumor-selective gene silencing profile of plofsome@*siFOSL1* (Figure S16C). These findings collectively demonstrate the favorable biosafety profile of plofsome@*siFOSL1* for potential therapeutic application.

In summary, the data demonstrate that plofsome@*siFOSL1* effectively inhibits tumor growth, extends survival in orthotopic GBM models, and exhibits excellent biocompatibility, underscoring its promise as a targeted therapeutic strategy for GBM.

## Discussion

The present study demonstrates that *FOSL1* transcriptionally upregulates *IKKα*, a pivotal kinase in NF-κB signaling, while IKKα-mediated phosphorylation stabilizes FOSL1, creating a self-amplifying positive feedback loop that drives persistent NF-κB activation and accelerates GBM progression. Furthermore, UCHL3 is identified as a key deubiquitinase required for maintaining FOSL1 stability. UCHL3 selectively cleaves K48-linked polyubiquitin chains from FOSL1, thereby preventing proteasome-mediated degradation and sustaining oncogenic signaling. Collectively, these findings define the FOSL1/IKKα/UCHL3 regulatory axis as a mechanistic driver of NF-κB-dependent tumorigenesis in GBM.

*FOSL1* has previously been implicated as a central mediator of GBM malignancy 48. Previous studies demonstrated that hypoxia-induced FOSL1 promotes GBM invasion, and pharmacologic inhibition of FOSL1 using HDAC inhibitor Entinostat results in significant tumor suppression in orthotopic xenograft models 49. Additionally, in the mesenchymal (MES) subtype of GBM, NF1 depletion was shown to modulate *FOSL1* expression through the RAS/MAPK signaling pathway, thereby sustaining proliferative, stem-like, and EMT-like phenotypes 49. Additional studies have reported that FOSL1 promotes UBC9-dependent SUMOylation of CYLD, thereby enhancing K63-linked polyubiquitination and activation of NF-κB signaling, ultimately facilitating the proneural-to-mesenchymal transition (PMT) in GSC 50. The current work confirms that *FOSL1* expression is significantly upregulated in GBM tissues and cell lines and that high FOSL1 expression correlates with adverse clinical outcomes. Functional assays confirm that *FOSL1* promotes proliferation, migration, invasion, and cell survival, highlighting its central role as a potent oncogenic driver in GBM progression. Moreover, the findings establish that *FOSL1*-mediated activation of NF-κB signaling constitutes a critical mechanism underlying its tumor-promoting effects. Despite these advances, the full scope of *FOSL1* regulatory activity in GBM—particularly its upstream activators, its dynamic interplay with other transcriptional networks, and potential compensatory pathways—remains incompletely understood and warrants further investigation.

IKKα, a catalytic subunit of the IKK complex, mediates NF-κB activation through both canonical and non-canonical signaling pathways 51, 52. IKKα has been shown to shuttle between the cytoplasm and nucleus while maintaining kinase activity within the nuclear compartment 53, 54. Although the role of IKKα/β in regulating NF-κB transcriptional activity has been extensively characterized, recent studies have identified additional phosphorylation substrates of IKKα/β that function independently of NF-κB, participating in a wide range of biological processes including cell growth, metabolism, apoptosis, cell cycle regulation, cell migration, and invasion 53-58. For instance, IKKβ stabilizes c-Fos, a member of the AP-1 transcription complex, *via* phosphorylation, thereby regulating cAMP-mediated cytokine production and contributing to the immunosuppressive actions of cAMP 59. Prior research has also reported that FOSL1 stability is closely associated with its phosphorylation state, which protects FOSL1 from ubiquitin-proteasome-mediated degradation 54, 60. In the present work, IKKα is identified as a transcriptional target of FOSL1, forming a reinforcing regulatory circuit wherein IKKα directly interacts with and phosphorylates FOSL1 at Ser265, thereby enhancing FOSL1 stability by preventing proteasome-dependent degradation.

The ubiquitin-proteasome system and the lysosomal pathway represent the major routes for protein turnover in eukaryotic cells 61. To elucidate the mechanism governing FOSL1 degradation, UCHL3 was identified as a critical deubiquitinating enzyme within the FOSL1-IKKα feedback loop. Prior studies indicate that UCHL3 preferentially cleaves extended ubiquitin chains and exhibits strong specificity for K48-linked ubiquitination 62. The present findings show that UCHL3 selectively removes K48-linked polyubiquitin chains from FOSL1 at lysine 156, thereby preventing proteasomal degradation and stabilizing the protein. Notably, IKKα-mediated phosphorylation of FOSL1 strengthened its association with UCHL3, as evidenced by diminished UCHL3-FOSL1 binding upon mutation of the phosphorylation site, supporting a phosphorylation-dependent recruitment mechanism that safeguards FOSL1 stability.

A recent study demonstrated that SR11302, a small-molecule inhibitor of FOSL1, significantly suppressed tumor growth in patient-derived xenograft models of head and neck squamous cell carcinoma 63. However, effective drug delivery to GBM remains challenging owing to the restrictive nature of the BBB 64-66. To address this limitation, a nanocapsule-based siRNA delivery system was engineered to enable targeted delivery of *siFOSL1* to GBM cells. This nanosystem successfully traversed the BBB, achieved potent FOSL1 gene silencing, and resulted in substantial inhibition of tumor growth as well as extended survival in GBM orthotopic mouse models. These findings highlight the therapeutic potential of targeting FOSL1 and underscore the promise of nanotechnology-enabled RNA interference as a precision treatment approach for GBM.

Despite these advances, several limitations warrant further investigation. First, although the functional interplay among FOSL1, IKKα, and UCHL3 has been delineated, the precise molecular mechanism through which IKKα enhances UCHL3 binding to FOSL1 remains incompletely understood. Immunoblotting indicates that IKKα phosphorylates FOSL1 within the C-DEST domain, while UCHL3 engages the bZIP region at the N-terminal. The manner in which C-DEST phosphorylation allosterically modulates UCHL3 interaction at this distal binding site remains unclear, including whether phosphorylation induces long-range conformational rearrangements that expose regulatory residues within the bZIP domain. Second, the long-term safety, immunogenicity, and potential off-target effects of the nanocapsule-based siRNA delivery system require rigorous evaluation across broader preclinical models. Third, while the current study defines the core *FOSL1*/IKKα/UCHL3 positive feedback loop as a driver of NF-κB-dependent tumorigenesis, the complete network of downstream translational and phenotypic consequences remains to be fully characterized. Comprehensive mapping of the effector landscape regulated by this axis will be critical for understanding the full biological and therapeutic implications of pathway suppression in GBM. Finally, our *in vivo* therapeutic evaluations were performed in immunodeficient models, which do not fully recapitulate the complex tumor-immune microenvironment of human glioblastoma. Future studies using immunocompetent models will be essential to assess the translational relevance and potential immunomodulatory effects of this strategy.

**Figure 8:**
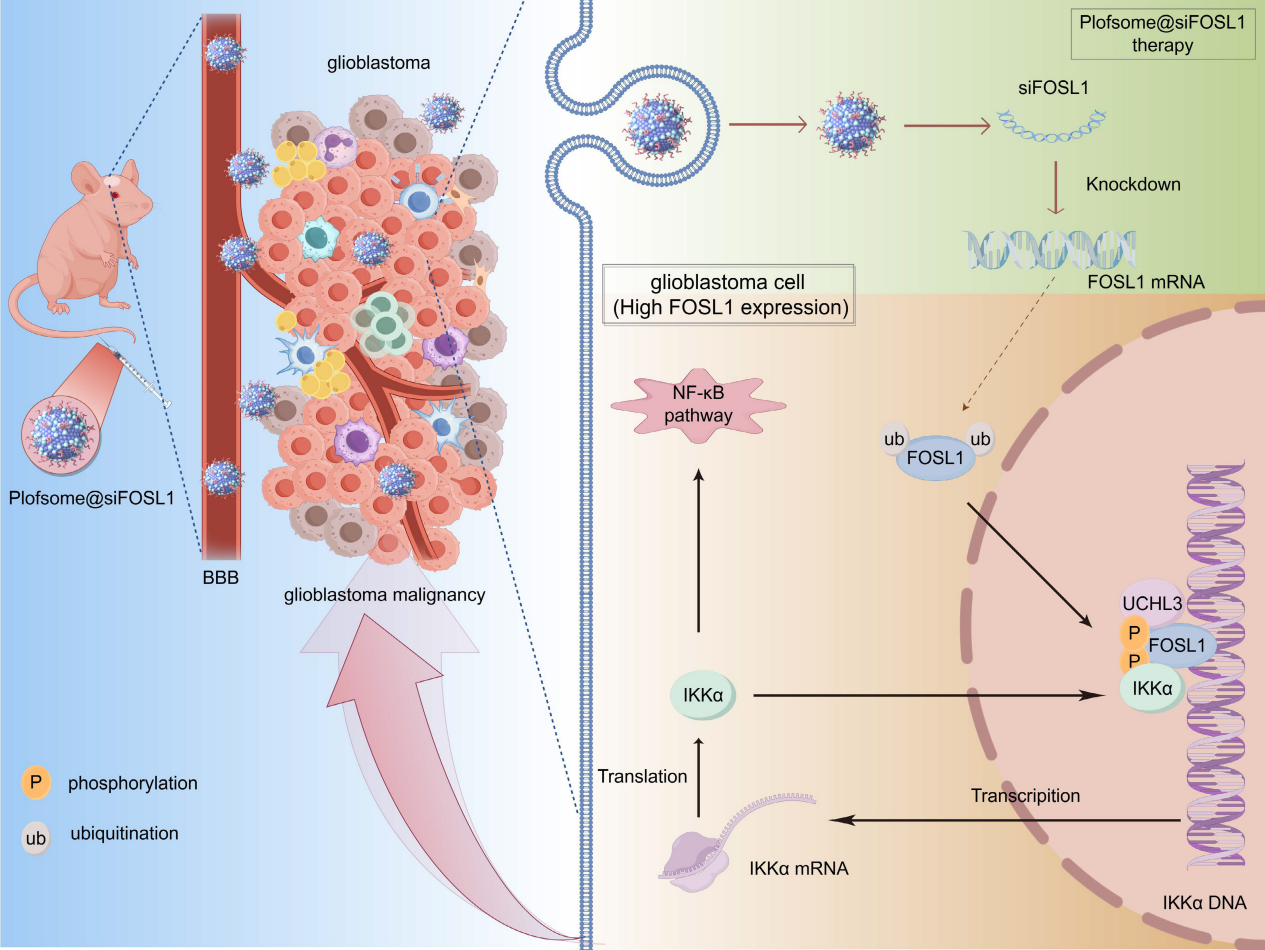
** Schematic diagram showing the regulatory mechanisms of FOSL1/IKKα/UCHL3 feedback loop mediated activation on NF-κB signaling in GBM.** Upon *FOSL1* upregulation in GBM, it transcriptionally activates IKKα, which in turn stabilizes FOSL1 by inhibiting its phosphorylation and ubiquitin-proteasomal degradation. Concurrently, UCHL3 enhances FOSL1 stability by cleaving K48-linked polyubiquitin chains. This FOSL1/IKKα/UCHL3 feedback loop drives NF-κB activation, promoting tumor progression, while plofsome @*sFOSL1* effectively suppresses GBM malignancy. Created with Figdraw.

In conclusion, the data from this study identify FOSL1 as a central regulator of GBM pathogenesis and propose the FOSL1/IKKα/UCHL3 feedback axis as a novel regulatory mechanism sustaining NF-κB signaling and malignant progression. The development of an *FOSL1*-targeted nanocapsule-based siRNA delivery system offers a promising strategy for precision therapy in GBM (Figure 8).

## Conclusion

This study demonstrates that FOSL1 drives glioblastoma malignancy through a previously unrecognized positive feedback loop involving IKKα and UCHL3. FOSL1 transcriptionally upregulates IKKα, which subsequently phosphorylates and stabilizes FOSL1 by inhibiting its ubiquitin-proteasomal degradation. UCHL3 further enhances FOSL1 stability by selectively removing K48-linked polyubiquitin chains. This self-sustaining regulatory axis maintains persistent NF-κB signaling activation, thereby promoting GBM proliferation, invasion, and survival. Furthermore, plofsome@siFOSL1 effectively targets FOSL1, suppresses tumor growth, and prolongs survival in orthotopic GBM models. Collectively, these findings identify the FOSL1/IKKα/UCHL3 loop as a key oncogenic mechanism and highlight its potential as a therapeutic target for GBM (Figure 8).

## Material and Methods

### Ethics

Approval for the use of patient-derived samples was granted by the Scientific Ethics Committee of The First Affiliated Hospital of Xi'an Jiaotong University, Xi'an, China (No. 2016-18). Written informed consent was obtained from all patients, and all experimental protocols were conducted in accordance with the principles outlined in the Declaration of Helsinki. A total of 50 glioma samples and 13 non-tumor brain specimens were collected from patients who underwent surgical resection between 2016 and 2021.

### Cox regression analysis

Gene expression profiles and relevant clinical data were extracted from the Chinese Glioma Genome Atlas (CGGA) and the Cancer Genome Atlas (TCGA). The analysis cohort comprised 225 primary glioblastoma samples from CGGA and 168 primary glioblastoma samples from TCGA with available RNA-seq data. Data preprocessing was performed in R Studio (version 4.0.0), including background correction, gene symbol unification, batch effect adjustment, and normalization. Univariate Cox regression analysis was applied to identify prognostic genes according to the following criteria: (1) both Likelihood P-value and Wald P-value were less than 0.05, and (2) hazard ratio (HR) indicating prognostic significance (HR > 1 and HR < 1). Additional refinement of candidate genes was performed using multivariate Cox regression *via* the R package *coxph*.

### Non-negative matrix factorization

To screen for prognostically relevant TFs, the filtered gene sets derived independently from TCGA GBM and CGGA GBM datasets were intersected with TFs curated from the Transcriptional Regulatory Relationships Unraveled by Sentence-based Text mining (TRRUST, https://www.grnpedia.org/trrust/downloadnetwork.php) database. Tumor heterogeneity and sample clustering based on prognostic TF signatures were evaluated using non-negative matrix factorization (NMF) methodology 67.

### Differential gene expression analysis

To minimize potential background noise, genes with an average expression level below 0.5 across all samples were excluded. Differentially expressed genes (DEGs) were identified using the *limma* package, with grouping based on the NMF classification 68. Expression differences were evaluated using log2 [Fold change] (log2FC) and adjusted P-values. Genes with log2FC > 2 and adjusted P < 0.05 were classified as upregulated, whereas those with log2FC < -2 and adjusted P < 0.05 were classified as downregulated.

### Gene set enrichment analysis

For GSEA based on FOSL1 expression, GBM samples were stratified into tertiles, with the top 33% designated as *FOSL1*-high and the bottom 33% as *FOSL1*-low. DEGs were ranked in descending order according to log2FC and analyzed in R Studio using the *clusterProfiler* package to identify significantly enriched pathways 69. A false discovery rate (FDR) of less than 0.05 with an absolute normalized enrichment score (NES) greater than 2.5 were used as criteria for enrichment.

### Cell lines and cell culture

The GBM cell lines U87, U251, LN229, and A172 were obtained from Servicebio Technology (Wuhan, China). U373 and the human astrocyte line SVGp12 were obtained from BNCC Technology (Shanghai, China), and hCMEC/D3 cells were obtained from SSRCC Technology (Shanghai, China). Cells were maintained in DMEM-F12 supplemented with 10% fetal bovine serum (FBS) and 1% penicillin-streptomycin, under a humidified atmosphere of 5% CO_2_ at 37 ℃. Culture medium was refreshed every 3 days.

### Construction and Transfection of plasmid, siRNA, and shRNA

siRNAs targeting *FOSL1* and *UCHL3*, along with corresponding negative controls, were obtained from TsingKe Biotechnology (Beijing, China). All siRNA sequences are listed in Supplementary Table S4. Plasmids encoding Flag-FOSL1, GFP-FOSL1, His-IKKα, Myc-UCHL3, GST-UCHL3, HA-Ubiquitin (WT, K0, K6, K11, K27, K29, K33, K48, K63), Myc-DUBs (VCPIP1, USP25, OTOD5, USP36), pGL4.11-IKKα-WT promoter luciferase reporter, and all corresponding mutants and deletion constructs were purchased from TsingKe Biotechnology (Beijing, China). The pRL-TK Renilla luciferase control plasmid was obtained from Beyotime Biotechnology (RG027, China). Transient transfections were performed using Lipo8000™ Transfection Reagent (C0533, Beyotime) according to the manufacturer's protocol.

Stable knockdown of *FOSL1*, *IKKα*, *IKKβ*, and *UCHL3* was established in GBM cell lines using shRNA designed and synthesized by TsingKe Biotechnology (Beijing, China). shRNA sequences are listed in Supplementary Table S4. Lentiviral particles were produced by co-transfecting HEK293T cells with shRNA constructs and packaging plasmids (pMD2.G and psPAX2) using Lipo8000™ transfection reagent. Viral supernatants were collected 48 h post-transfection, filtered through a 0.45-µm membrane, and used to infect GBM cells in the presence of 5 µg/ml Polybrene (C0351, Beyotime). Stable knockdown cells were selected using 1 µg/ml puromycin (ST551, Beyotime) for 72 h.

For overexpression experiments, lentiviruses encoding *FOSL1* and *IKKα* were obtained from Genechem Biotechnology (Shanghai, China), whereas lentiviruses encoding luciferase were obtained from Wz Biosciences (Shandong, China). Stable overexpression cell lines were generated according to the manufacturers' protocols.

### Cell proliferation assay

The proliferative capacity of GBM cells was assessed using the CCK-8 (C0037, Beyotime), colony formation assays, and EdU incorporation assays (C0075, Beyotime). For the CCK-8 assay, cells were resuspended and seeded into 96-cell plates at a density of 2 × 10^3^ cells/100 μL per well and cultured at 37 °C with 5% CO₂ for 24, 48, 72, and 96 h. Following incubation with CCK-8 reagent for 1 h at 37 °C, absorbance at 450 nm was measured using a microplate reader (BioTek) to determine cell viability.

For colony formation assays, pretreated cells were seeded into 6-well plates under indicated experimental conditions and maintained for 14 days to allow colony formation. Colonies were fixed with methanol, stained with 0.1% crystal violet solution (G1014, Servicebio Technology), and quantified to determine clonogenic capacity.

For EdU assays, pretreated cells were seeded into 96-well plates at a density of 1 × 10^4^ cells per well and cultured for 24 h. Cells were subsequently incubated with EdU reagent for 2 h at 37 °C, fixed with 4% paraformaldehyde, and counterstained with Hoechst 33342 for 10 min. Images were acquired using a fluorescence microscope (Olympus), and the proportion of EdU-positive cells was calculated to determine the proliferation index. All assays were performed in triplicate.

### Cell invasion and migration assays

Cell invasion was assessed using 8-µm pore Transwell inserts (Corning). The upper chambers were precoated with Matrigel matrix (356234, Corning) and incubated at 37 °C for 1 h to allow gelation. Pretreated GBM cells were seeded into the upper chamber at a density of 5 × 10⁴ cells/200 µL. The lower chamber was filled with 750 µL of complete growth medium containing 10% FBS. After 12 h of incubation at 37 °C, invaded cells on the lower membrane surface were fixed in methanol for 30 min, stained with 0.1% crystal violet, washed with phosphate-buffered saline (PBS), and imaged using a digital microscope (Olympus). Non-invading cells remaining on the upper membrane surface were gently removed with a cotton swab.

Cell migration was evaluated using a wound healing assay. Pretreated cells were seeded into 6-well plates and cultured to 90-95% confluence. A uniform linear scratch was generated in the cell monolayer using a sterile 10 µL pipette tip. Wound closure was recorded at 0 and 24 h post-scratching using a digital microscope (Olympus). The wound margins were mapped, and the relative migration distance was quantified using ImageJ software. All experiments were conducted in triplicate.

### Immunoblot analysis

Total protein was extracted using RIPA lysis buffer (P0013B, Beyotime) supplemented with protease inhibitor cocktail (HY-K0010, MCE) and phosphatase inhibitor cocktails (HY-K0022, HY-K0023, MCE). Cytoplasmic and nuclear protein fractions were isolated using the Nuclear and Cytoplasmic Protein Extraction Kit (P0027, Beyotime). Protein concentration was determined using a BCA protein quantification kit (E112, Vazyme). Protein samples were separated by SDS-PAGE and transferred onto PVDF membranes. After blocking with 5% non-fat milk in TBST for 1 h at room temperature, membranes were incubated with primary antibodies overnight at 4 °C. After washing with TBST, the membranes were incubated with secondary antibodies for 1 h at room temperature. Protein bands were visualized using an Ultra High Sensitivity ECL Kit (HY-K1005, MCE), and chemiluminescence signals were captured using a digital imaging system. A complete list of antibodies is provided in Supplementary Table S1.

### Co-immunoprecipitation (Co-IP) assay

For Co-IP assays, total cellular protein was extracted using IP lysis buffer (G2038, Servicebio Technology). Cell lysates were incubated with specific antibodies or control IgG overnight at 4 °C. Immune complexes were captured using Protein A/G magnetic beads and incubated for an additional 4 h at 4 °C. Bound proteins were eluted by boiling in 1× SDS-PAGE loading buffer for 5 min and subsequently subjected to immunoblot analysis.

### *In vitro* kinase assay

For *in vitro* kinase assay, 20 µg of recombinant FOSL1 (Ag25788, Proteintech) and 10 µg of recombinant IKKα (P5564, Abnova) were incubated in a 30 µl reaction mixture containing 20 µl of kinase buffer (#9802, Cell Signaling Technology) and 200 µM ATP. The kinase reaction was carried out at 30 °C for 2 h and terminated by adding 1× SDS-PAGE loading buffer, followed by boiling at 95 °C for 10 min.

### Quantitative RT-PCR

Total RNA was extracted using the Cell RNA Isolation Kit (RC102, Vazyme) and reverse-transcribed into cDNA using RevertAid Master Mix (M16325, Thermo). Quantitative real-time PCR was performed using gene-specific primers, with GAPDH serving as the internal reference. Primer sequences are listed in Supplementary Table S2. Gene expression was quantified using the 2^-ΔΔCt^ method, normalized to GAPDH. All reactions were conducted in triplicate.

### Immunofluorescence staining

Pretreated GBM cells were seeded onto confocal imaging dishes, fixed with 4% paraformaldehyde for 30 min, and permeabilized using 0.5% Triton X-100 (G1204, Servicebio) for 20 min. Cells were blocked with 5% BSA in PBS for 1 h and incubated with primary antibodies overnight at 4 °C. After washing with PBS, fluorophore-conjugated secondary antibodies were applied for 1 h at 37 °C, followed by nuclear staining with DAPI for 10 min at room temperature. Images were captured using a confocal laser scanning microscope (Olympus).

### H&E and IHC staining

Formalin-fixed, paraffin-embedded (FFPE) tumor tissues from clinical patients and brain tissues from euthanized nude mice were sectioned coronally at a thickness of 4 µm. H&E and IHC staining were performed by Yike Biological Technology (Shaanxi, China). For H&E staining, tissue sections were deparaffinized, stained with hematoxylin for 3-8 min, and counterstained with eosin for 1-3 min.

For IHC staining, deparaffinized slides were treated with 0.3% methanol-hydrogen peroxide to quench endogenous peroxidase activity and subsequently blocked with 5% BSA for 1 h. Slides were then incubated with primary antibodies overnight at 4 °C, followed by sequential incubation with biotinylated goat anti-rabbit/mouse IgG and streptavidin-HRP at room temperature. Chromogenic development was performed using DAB, and nuclei were counterstained with hematoxylin.

FOSL1 expression was quantified using the H-score system. Staining intensity was graded on a scale of 0 to 3, where 0 = negative, 1 = weak, 2 = moderate, and 3 = strong. The percentage of tumor cells exhibiting each intensity level was recorded, and the H-score was calculated as:

H-score = (0 × % of cells with intensity 0) + (1 × % of cells with intensity 1) + (2 × % of cells with intensity 2) + (3 × % of cells with intensity 3), yielding values ranging from 0 to 300. All immunohistochemical slides were independently evaluated by two experienced pathologists, who were blinded to the clinical data. Discrepant assessment scores were jointly reviewed to reach a consensus.

### Flow cytometry analysis

Flow cytometry was used to assess apoptosis. The Annexin V-PE/7-AAD Apoptosis Detection Kit (A213, Vazyme) was used to determine the proportions of apoptotic cells under different treatments. Cells were collected, washed 3 times with PBS, and incubated with Annexin V-PE and 7-AAD for 10 min at room temperature in the dark. Samples were analyzed using a flow cytometer (BD Biosciences). All assays were performed in triplicate.

### RNA sequencing

RNA extraction, library construction, sequencing, and data analysis was conducted by Biomarker Technologies, Beijing, China.

### Chromatin immunoprecipitation (ChIP)

ChIP assays were performed using the SimpleChIP^®^ Enzymatic Chromatin IP Kit (9003, CST). Briefly, cells were fixed with 37% formaldehyde for 10 min, and crosslinking was quenched with glycine. Chromatin was fragmented using micrococcal nuclease digestion followed by sonication. Lysates were incubated with primary antibodies overnight at 4 °C, followed by incubation with protein G magnetic beads for 2 h at 4 °C. DNA was eluted and purified from both input and IP fractions. ChIP-PCR and ChIP-qPCR were conducted using promoter-specific primers designed to amplify target promoter region. Primer sequences are provided in Supplementary Table S3. The resulting PCR products were visualized by agarose gel electrophoresis.

For ChIP-seq analysis, both input and IP DNA were submitted for high-throughput sequencing, performed by Igenbool Biotechnology (Wuhan, China). Raw sequencing data were processed on the Illumina sequencing platform, and quality assessment was performed using FastQC (v0.11.5), followed by preprocessing to retain high-confidence sequences. Reads were aligned to the human reference genome (GHRCh38_p14) using BWA (v0.7.15-r1140) with optimized parameters. Peak calling and annotation for identification of FOSL1 binding sites were conducted using the R package *chipseeker*. Genomic data visualization and integrative inspection of binding regions were performed using IGV (v16.2). The JASPAR database (https://jaspar.genereg.net/) was used to identify and validate predicted FOSL1 binding motifs within the *IKKα* promoter region.

### Luciferase activity assay

Luciferase activity was measured using the Dual Luciferase Reporter Gene Assay Kit (RG027, Beyotime). HEK293T cells were transfected with wild-type or mutant promoter luciferase constructs, and reporter activity was measured 48 h post-transfection using a microplate reader (BioTEK). Renilla luciferase served as the internal control for normalization.

### DNA pull-down assay

Recombinant IKKα protein, synthesized with or without site-specific biotin conjugation (TsingKe Biotechnology, Beijing, China), was immobilized on streptavidin-coated beads by incubation for 30 min. The beads were subsequently incubated with nuclear protein extracts for 1 h to allow protein complex formation. After three washes with binding buffer, bound protein complexes were eluted in 100 μL of 1× SDS loading buffer at 65 °C for 10 min and analyzed by SDS-PAGE.

### Mass spectrometry analysis

To identify FOSL1-interacting proteins, cell lysates from U87 cells were subjected to immunoprecipitation using anti-FOSL1 antibody and Protein A/G magnetic beads. Immunoprecipitated complexes were submitted to Bioprofile Biotechnology (Shanghai, China) for LC-MS/MS-based proteomic analysis.

### GST pull-down assay

For GST pull-down assays, recombinant GST-FOSL1 (P01, Abnova) was incubated with recombinant His-IKKα (TsingKe Biotechnology) or His-UCHL3 (Ag32925, Proteintech) together with BeyoGold™ GST-tag Purification Resin (P2251, Beyotime) in protein binding buffer overnight at 4 °C. After extensive washing with elution buffer, bound proteins were eluted by boiling in 1×SDS-PAGE loading buffer for 10 min, followed by immunoblot analysis.

### Preparation of plofsome@*siFOSL1*

For synthesis of the block copolymer PPPM, the previously reported amino-functionalized polymer was used as the starting reagent. ^1^H-pyrazole-1-carboxamidine hydrochloride and N, N-diisopropylethylamine (DIEA) were added in DMSO, and the mixture was stirred at room temperature for 24 h. The reaction mixture was subsequently dialyzed against water, and the solvent was removed by evaporation. Residual solid was dissolved in a small volume of methanol, followed by slow addition of ethyl ether to precipitate the final polymer.

For the preparation of plofsome@*siFOSL1*, siRNA (100 μg/mL) and PPPM (1.5 mg/mL) were mixed and stirred at room temperature for 4 h. The complex was purified by dialysis against PBS (molecular weight cutoff of 10 kDa). Successful assembly of plofsome@*siFOSL1*was confirmed by DLS and transmission electron microscopy (TEM), and samples were stored at 4 °C until use.

### Stability assessment of plofsome@*siFOSL1*

The stability of naked siRNA versus plofsome@*siFOSL1* was evaluated by agarose gel electrophoresis. Samples were treated with 1 mg/ml RNase A (ST578, Beyotime) for 0, 5 min, 30 min, 1 h, 3 h, and 6 h at 37 °C prior to electrophoretic analysis.

### Lysosome escape

U87 cells were incubated with C6-labeled plofsome@*siNC* for 1 h or 4 h, followed by replacement with fresh medium and staining with LysoTracker Red. Cells were then fixed with 4% paraformaldehyde and counterstained with DAPI. Fluorescence images were acquired using a confocal microscope (Olympus).

### *In vitro* BBB model

Transwell inserts with 0.4 μm pore size (12-mm Transwell, Corning) were uniformly coated with Matrigel basement membrane matrix (254234, Corning). Immortalized human brain microvascular endothelial cells (HCMEC/D3) were seeded into the upper chamber. When the transendothelial electrical resistance (TEER) exceeded 200 Ω cm^2^, the monolayer was considered suitable for permeability experiments. U87 cells were seeded into the lower chamber. To assess BBB penetration of plofsome@*siFOSL1*, C6-labeled nanocomplex was added to the upper chamber and incubated at 37 °C. After 1 and 4 h, U87 cells from the lower chamber were collected, fixed with 4% paraformaldehyde, and counterstained with DAPI. Fluorescence imaging was performed using a confocal microscope (Olympus).

### *In vitro* cytotoxicity assay

The cytotoxicity of plofsome@*siNC* and plofsome@*siFOSL1* was evaluated using the CCK-8 assay. U87 cells were seeded into 96-well plates at a density of 1 × 10³ cells/well and treated with a concentration gradient of plofsome@*siNC* or plofsome@*siFOSL1* (1-1000 µM) for 72 h. After treatment, CCK-8 reagent was added to each well and incubated for 1 h at 37 °C. Absorbance at 450 nm was measured on a microplate reader (BioTek). The half-maximal inhibitory concentration (IC₅₀) was calculated using GraphPad Prism 10.1.2.

### Orthotopic GBM mouse model

Male BALB/c nude mice (6-8 weeks old) were obtained from Shanghai Model Organisms Center, Inc. Mice were anesthetized using isoflurane (3% for induction and 1% for maintenance), and 1 × 10⁵ U87 cells were stereotactically injected into the right striatum (coordinates: 2 mm lateral, 1 mm anterior to the bregma; depth: 3.5 mm). For U87-Luc models, intracranial tumor engraftment was confirmed by the IVIS imaging system 10 days post-injection. Tumor-bearing mice were randomly assigned to experimental groups (n = 9 per group). All animal procedures were approved by the Animal Ethics Committee of Xi'an Jiaotong University (No. 2021-695) and were conducted in compliance with the NIH Guide for the Care and Use of Laboratory Animals. All *in vivo* studies complied with the Animal Research: Reporting of *In Vivo* Experiments (ARRIVE) reporting guidelines.

### *In vivo* biodistribution of plofsome@*siNC*

Upon confirmation of intracranial tumor formation by *In Vivo* Imaging System (IVIS), mice received a tail vein injection with plofsome@*siNC*/IR780 (2 mg/kg). Whole-body fluorescence was recorded at 1, 3, 6, 12, 24, and 48 hs post-injection using the IVIS imaging system. At 24 hs post-injection, mice were euthanized, and major organs (heart, liver, spleen, lung, kidney, and brain) were collected for *ex vivo* fluorescence imaging. Fluorescence intensities were quantified to assess biodistribution.

### *In vivo* antitumor efficacy of plofsome@*siFOSL1* in U87-Luc orthotopic model

U87-Luc tumor-bearing nude mice were stratified by baseline bioluminescence intensity and randomized into three treatment groups (n = 9 per group). Mice received intravenous tail-vein injections of PBS, plofsome@*siNC*, or plofsome@*siFOSL1* (2 mg/kg) every two days. Tumor progression was monitored using the IVIS imaging system at days 0, 5, 10, and 15 following treatment initiation. On day 15, three mice per group were euthanized for the collection of brain tissues for histopathological analysis, including H&E and IHC staining. Survival curves were generated and analyzed using the Kaplan-Meier methodology.

### *In vivo* biosafety assessment

To evaluate the biosafety profile of plofsome@*siFOSL1 in vivo*, total protein was extracted from the brain and major organs (liver, lung, and kidney) to examine target-gene silencing efficiency and potential off-target effects.

Systemic toxicity was preliminarily evaluated by hematological and histopathological examinations. Liver function indices—including alanine aminotransferase (ALT), aspartate aminotransferase (AST), albumin (ALB), and total bilirubin (TBIL)—and renal function markers—blood urea nitrogen (BUN) and creatinine (CREA)—were measured. Concurrently, histological sections of major organs were examined by H&E staining to assess tissue morphology and structural integrity.

### Drugs

For protein stability assays, cells were treated with cycloheximide (CHX, 100 µM; HY-12320, MCE) and harvested at 0, 3, and 6 h post-treatment. To assess ubiquitination levels, cells were treated with the proteasome inhibitor MG132 (25 µM, HY-13259, MCE) for 8 h to prevent ubiquitin-mediated protein degradation. Activation of NF-κB signaling was achieved by treating GBM cells with TNFα (200 ng/mL, P5322, Beyotime) for 72 h. Inhibition of UCHL3 activity was performed by treating cells with TCID (10 µM, HY-18638, MCE) for 24 h, whereas ERK2 activity was inhibited by using ulixertinib (10 µM, HY-15816, MCE) for 24 h.

### Statistical analysis

All quantitative data are presented as mean ± standard deviation (SD), with the number of independent replicates indicated in the corresponding figure legends. Statistical differences between two groups were assessed by two-tailed Student's *t*-tests, and comparisons among multiple groups were performed using one-way analysis of variance (ANOVA). Survival data were analyzed using the Kaplan-Meier method, with statistical significance evaluated by log-rank testing. Statistical analyses were conducted using GraphPad Prism 10.1.2 or R Studio. A two-sided *P*-value of < 0.05 was considered statistically significant unless otherwise specified.

## Supplementary Material

Supplementary figures and tables.

## Abbreviations

GBM: glioblastoma
FOSL1: FOS-like antigen 1
IKKα: inhibitor of nuclear factor kappa-B kinase subunit alpha
UCHL3: ubiquitin C-terminal hydrolase L3
Plofsome@*siFOSL1*: *FOSL1* via a novel nano-based siRNA delivery system
TFs: transcription factors
bZIP: basic leucine zipper domain
EMT: epithelial-to-mesenchymal transition
NF-κB: nuclear factor kappa-light-chain-enhancer of activated B cells signaling pathway
IκB: inhibitor of kappa B
ChIP: chromatin immunoprecipitation
co-IP: co-immunoprecipitation assays
DUBs: deubiquitinating enzymes
C6: Coumarin 6
H&E: hematoxylin-eosin staining
MES: mesenchymal
PDX: patient-derived xenograft
CGGA: Chinese Glioma Genome Atlas
IHC: immunohistochemical staining
GSEA: gene set enrichment analysis
PCA: principal components analysis
WT: wild-type
