# Supplementary material for: Targeting the FOSL1/IKKα positive feedback loop attenuates glioblastoma malignancy via suppression of NF-κB signaling
Source: Theranostics. 2026 Jan 1;16(7):3338–62. doi: 10.7150/thno.121770 (PMC12846734; doi:10.7150/thno.121770)
Supplement: Supplementary file 1 — Supplementary figures and tables. [file thnov16p3338s1.pdf]

Supplementary Materials for

Targeting FOSL1/ IKKα positive feedback loop attenuates malignancy of glioblastoma via suppression of NFKB signaling

Beichen Zhang†, Hao Tian†, Haoyu Zhou†, Yichang Wang, Ke Gao, Yiyang Cao, Mingjing Zhou, Maode Wang*, Wei Wu*, Jia Wang*

Correspondence to: jiawang_xjtu@163.com

This PDF file includes:

Figures. S1 to S16
Tables S1 to S4

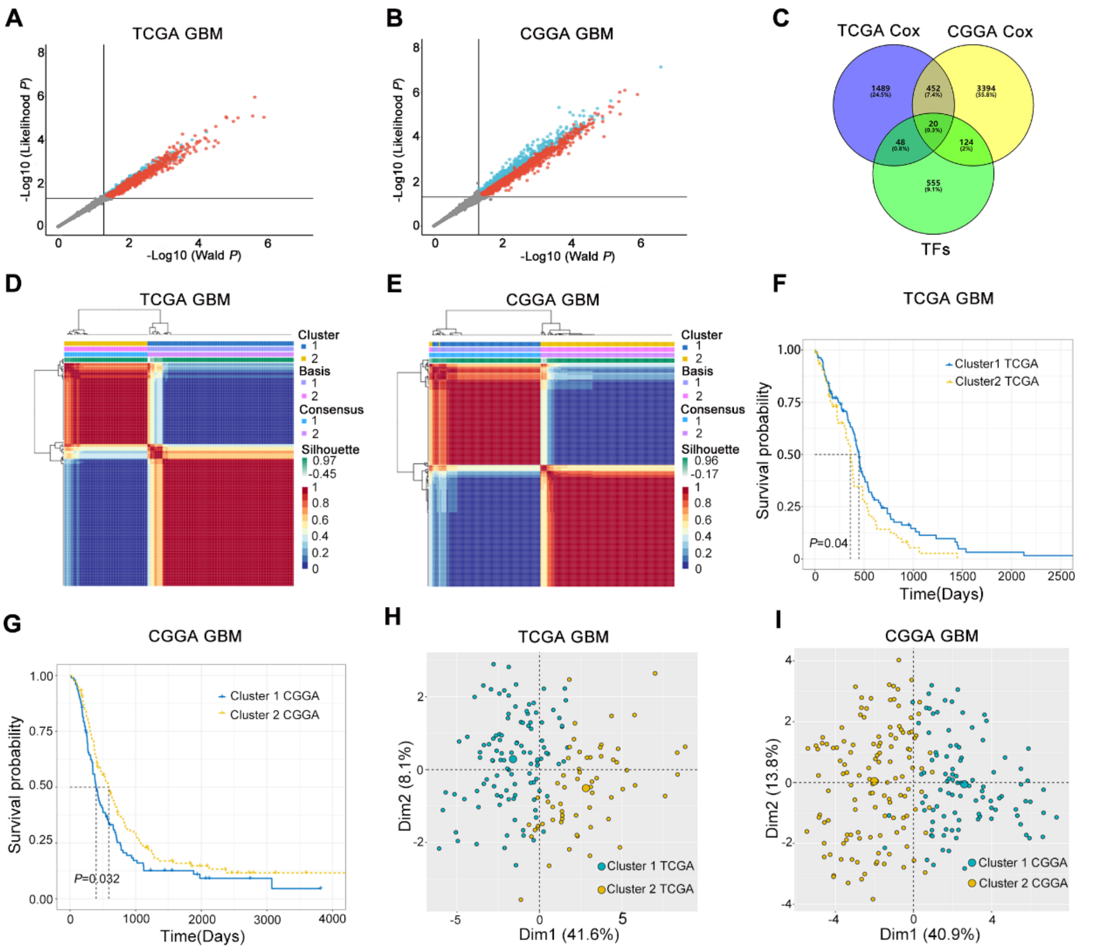

Figure S1. GBM were stratified into distinct subgroups base on the expression profiles of prognostic transcription factors (TFs).

A and B, Cox regression analysis was performed to identify prognostic genes using mRNA expression profiles from the TCGA and CGGA GBM datasets. Red dots indicate genes with a hazard ratio (HR) > 1, while blue dots represent genes with HR < 1; both were considered statistically significant with P-values (Likelihood P-value and Wald P-value) < 0.05. Grey dots denote genes without statistical significance. **C,** A Venn diagram was used to intersect the human TFs gene list with the prognostic genes to identify prognostic TFs. **D and E,** NMF clustering algorithm was applied to analyze the expression matrices of the 20 prognostic TFs in the TCGA and CGGA GBM datasets. **F and G,** Kaplan-Meier analysis was performed to compare survival outcomes between Cluster 1 and Cluster 2 in TCGA and CGGA GBM datasets, derived from the NMF clustering algorithm. All comparisons showed statistically significant differences (P < 0.05, log-rank test). **H and I,** PCA analysis were conducted to evaluate the effectiveness of the subgroup classification in the TCGA and CGGA GBM datasets, where Dim1 and Dim2 represent the first and second principal components, respectively.

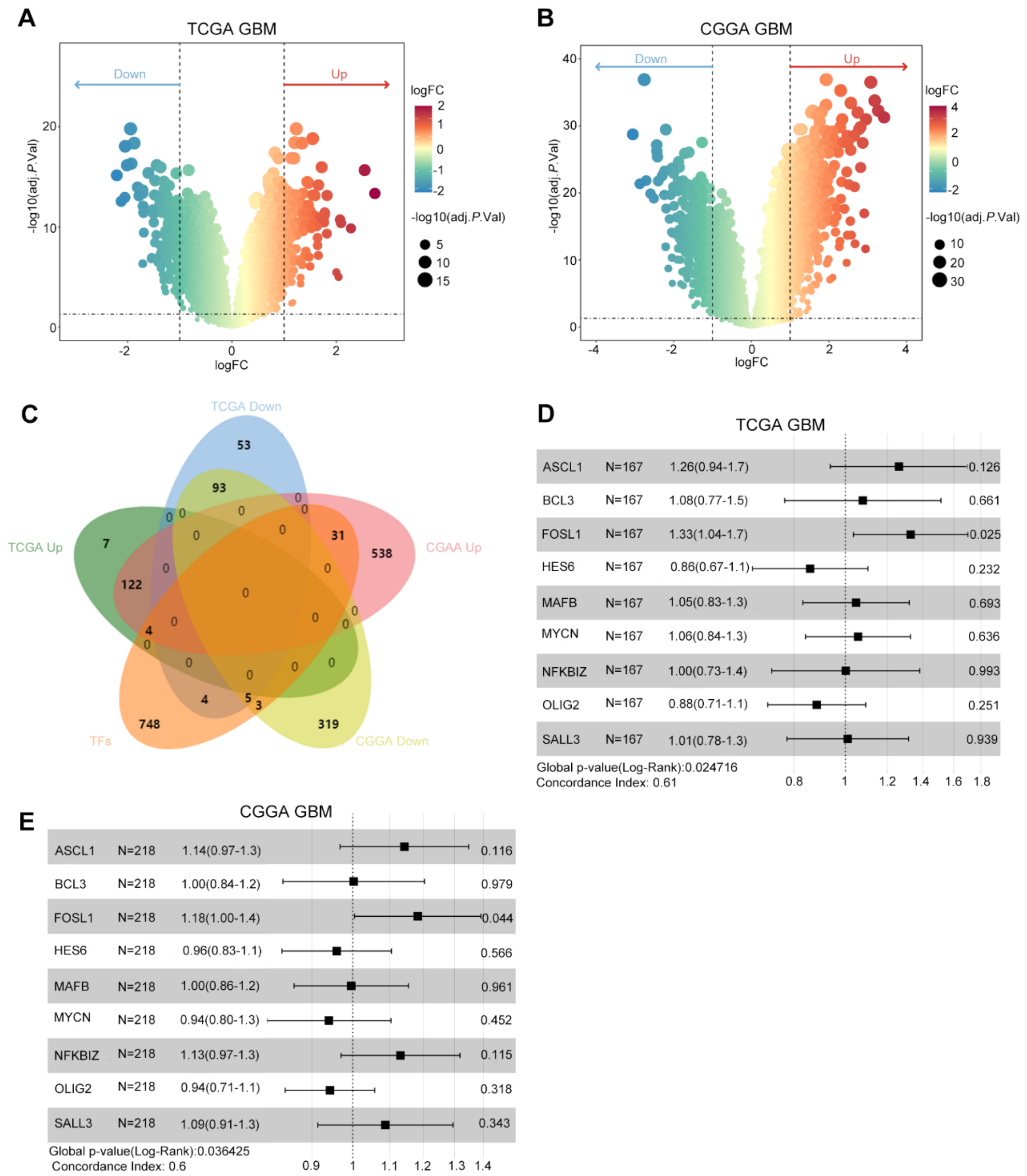

Figure S2. *FOSL1* overexpression defined a molecular signature in GBM.

A and B, Identification of differentially expressed genes (DEGs) between Cluster 1 and Cluster 2 subgroups through comparative analysis of TCGA and CGGA datasets. **C,** Integration of DEGs with transcription factor profiles using Venn diagram analysis. **D and E,** Prognostic significance evaluation of candidate transcription factors through multivariate Cox regression analysis, presented as forest plots.

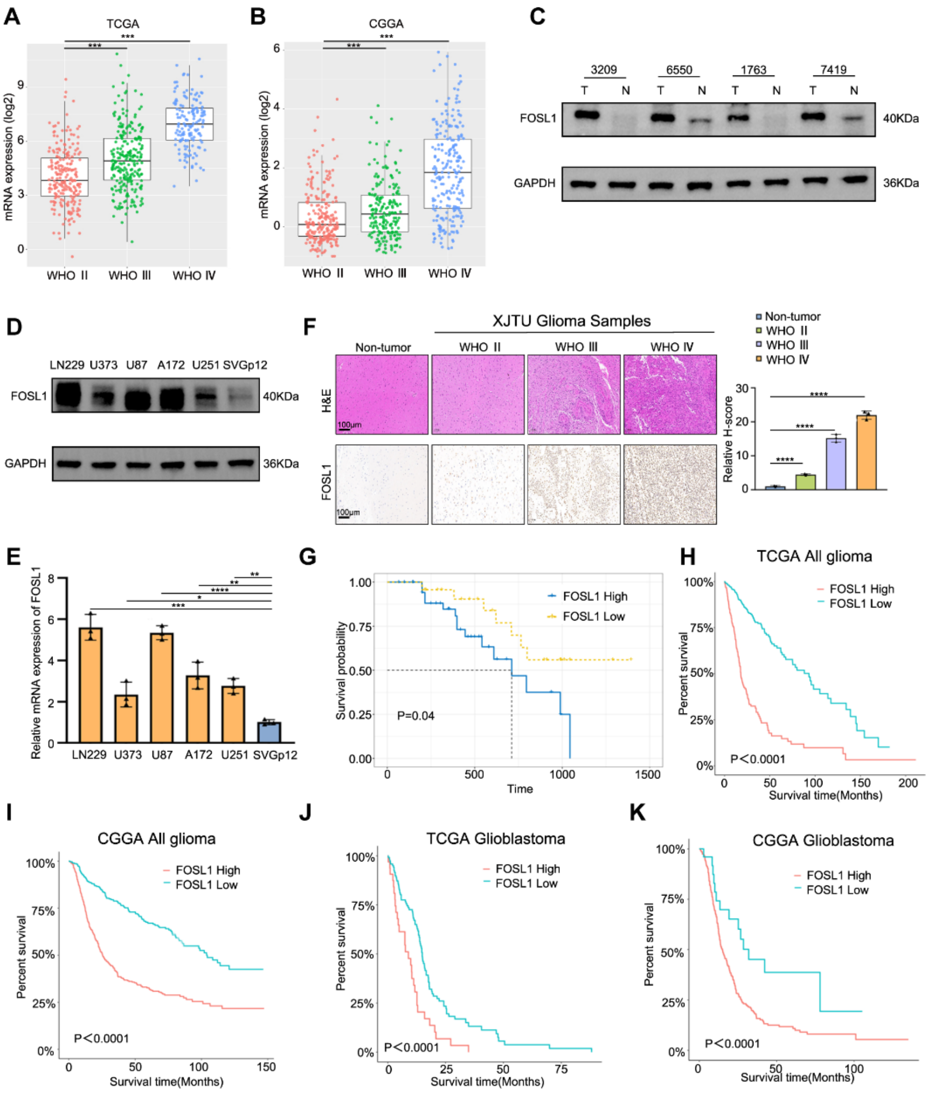

Figure S3. Elevated expression of FOSL1 was associated with poor prognosis in GBM patient.

A and B, the mRNA expression levels of *FOSL1* in gliomas were stratified according to the WHO classification using the GlioVis database (***$P < 0.001$, HSD test). **C,** immunoblot analysis was performed to assess FOSL1 expression in GBM tumor tissues compared to non-tumor tissues. **D and E,** qRT-PCR and immunoblot analysis were conducted to evaluate FOSL1 expression in GBM cell lines and human astrocyte cell line (SVGp12) cells. (**$P < 0.01$, n = 3, independent sample *t* test). **F,** Representative immunohistochemistry (IHC) staining of FOSL1 in glioma and non-tumor tissues using tissue microarray (TMA) (top: H&E staining; bottom: IHC staining of FOSL1). Quantitative analysis using the H-score system was performed for IHC evaluation. Scale bars, 100 μm. **G,** Kaplan-Meier analysis was performed based on the high immunohistochemical score (IHS) of *FOSL1* expression ($P = 0.04$). **H-K,** Kaplan-Meier analysis was conducted for *FOSL1* expression in all glioma (**H and I**) and GBM patient samples (**J and K**) using the GlioVis dataset (all $P < 0.0001$, log-rank test). **L-O,** Kaplan-Meier survival analysis of GBM patients stratified by *FOSL1* expression levels in subgroups defined by IDH status (L and M) and sex (N and O). Analyses

were performed using the GlioVis database (all log-rank $P < 0.0001$). GAPDH was used as the loading control for normalization. Data shown as mean ± SD. The immunoblotting experiments were repeated three times with similar results.

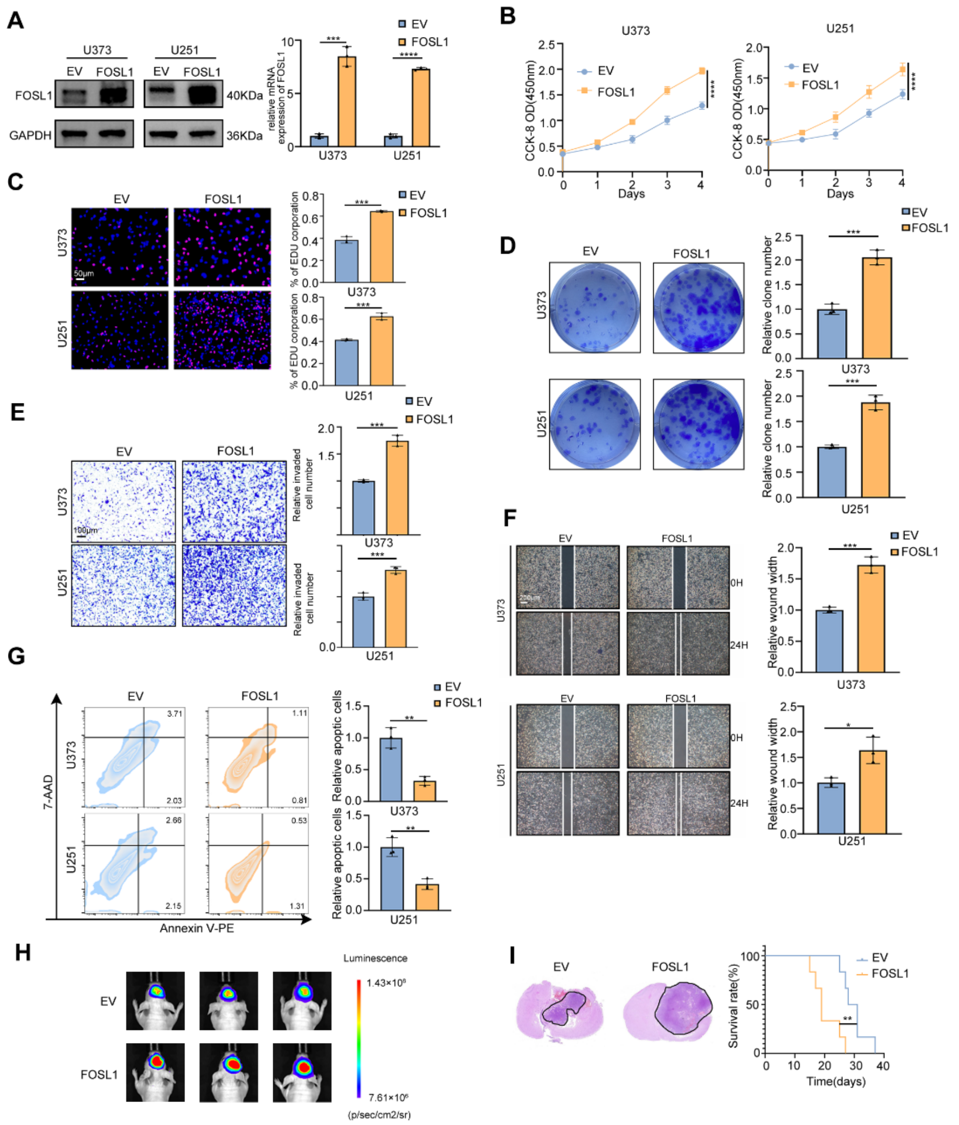

Figure S4. Overexpression of FOXL1 enhanced the malignancies of GBM cells.

A, FOSL1 overexpression efficiency was confirmed by qPCR (n = 3, with independent sample *t* test) and immunoblot analysis in U373 and U251 cells. **B-D,** the effect of FOSL1 overexpression on cell proliferation was evaluated by CCK-8 assays (n = 3, with one-way ANOVA test, **B**), EDU assays (n = 3, with independent sample *t* test, Scale bars, 50 μm **C**), colony formation assays (n = 3, with independent sample *t* test, **D**). **E,** Cell Matrigel invasion assays was performed to evaluate cell invasion in GBM cells following FOSL1 overexpression (n = 3, with independent sample *t* test). Scale bars, 100 μm. **F,** Wound-healing assays was performed to assess cell migration in GBM cells following FOSL1 overexpression (n = 3, with independent sample *t* test). Scale bars, 200 μm. **G,** Flow cytometry-based apoptosis analysis was used to evaluate cell apoptosis in GBM cells following FOSL1 overexpression (with independent sample *t* test). **H and I**, Representative bioluminescent images (**H**), H&E staining and Kaplan-Meier analysis (**I**) (n = 6 in each group, with log-rank test) of U373 orthotopic xenograft nude mice following FOSL1 overexpression. GAPDH was used as the loading control for normalization. * $P < 0.05$, ** $P < 0.01$, *** $P < 0.001$, **** $P < 0.0001$. Data shown as mean ± SD. The immunoblotting experiments were repeated three

times with similar results.

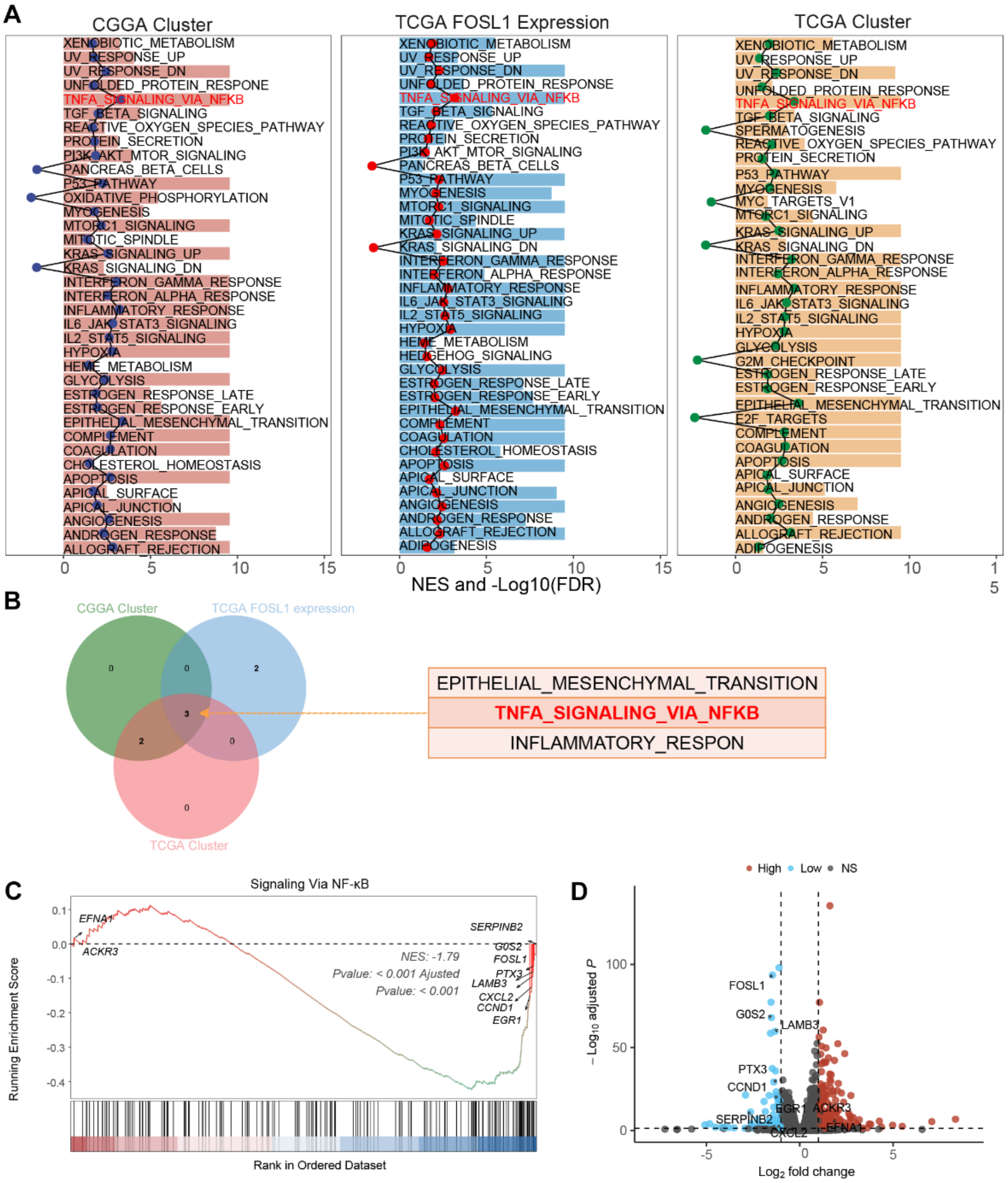

Figure S5, related to Figure 2. FOSL1 promoted malignancies of GBM through activation of NF-κB signaling pathway.

A, Gene Set Enrichment Analysis (GSEA) was used to identify the significantly enriched signaling pathways in TCGA and CGGA datasets. **B,** A Venn diagram was used to intersect the top 5 ranked pathways (|NES| > 2.5 and FDR < 0.05) from the 3 independent GSEA analyses. **C,** GSEA plot demonstrating a significant negative enrichment of the NF-κB signaling pathway gene set in *FOSL1*-knockdown glioma cells compared to control cells (NES: -1.79, FDR < 0.001). **D,** Volcano plot showing differentially expressed genes following *FOSL1* knockdown.

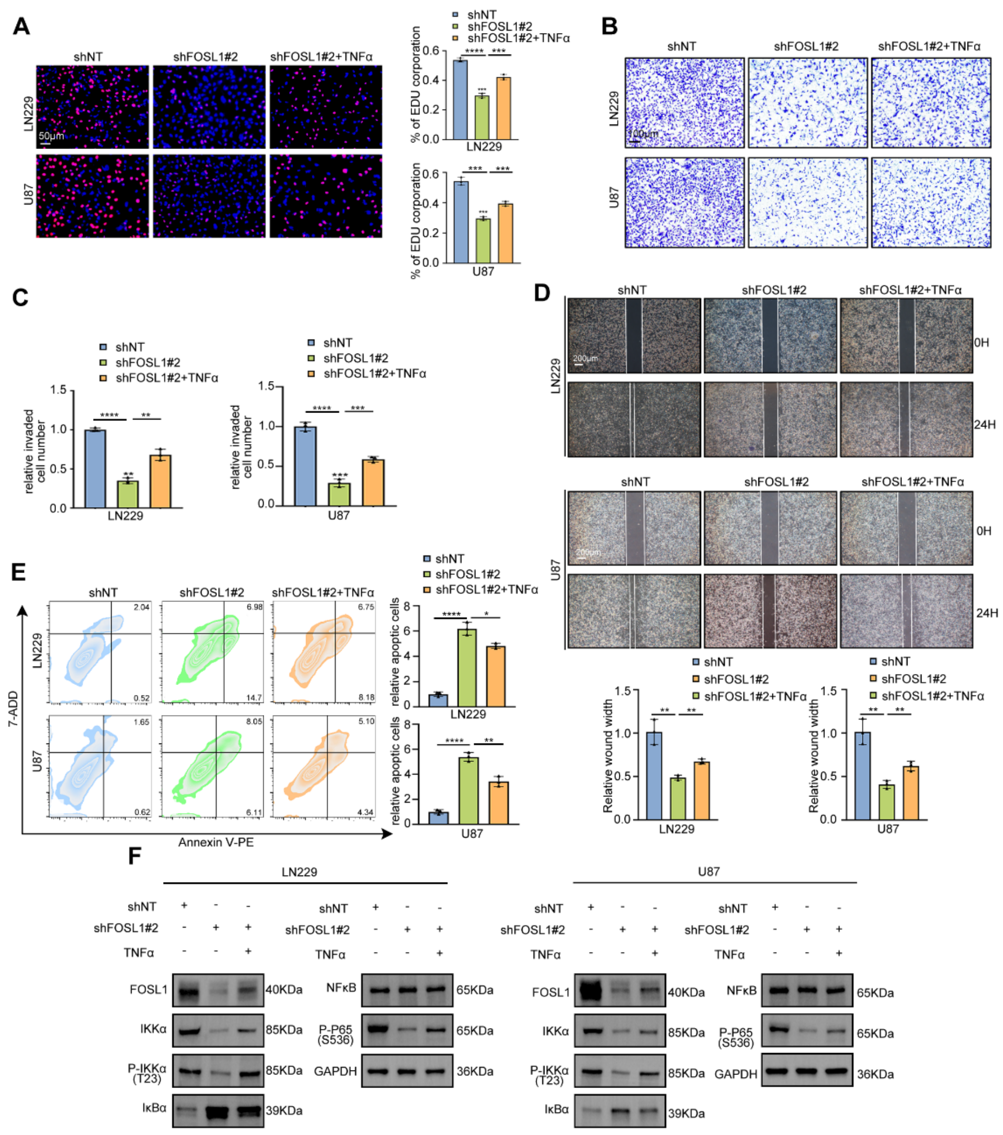

Figure S6, related to Figure 2. FOSL1 promoted malignancies of GBM through activation of NF-κB signaling pathway. A, EDU assay was employed to evaluate cell proliferation in LN229 and U87 cells following FOSL1 knock-down, with or without TNFα (200 ng/ml, 72h) treatment (n = 3, with independent sample *t* test). Scale bars, 50 µm. **B and C**, Cell Matrigel invasion assays were performed to evaluate cell invasion in LN229 and U87 cells following FOSL1 knock-down, with or without TNFα (200 ng/ml, 72 h) treatment (n = 3, with independent sample *t* test). Scale bars, 100 µm. **D,** Wound-healing assay was conducted to assess cell migration in LN229 and U87 cells following FOSL1 knock-down, with or without TNFα (200 ng/ml, 72 h) treatment (n = 3, analyzed by independent sample *t* test). Scale bars, 200 µm. **E,** Flow cytometry-based apoptosis analysis was used to evaluate cell apoptosis in LN229 and U87 cells following FOSL1 knock-down, with or without TNFα (200 ng/ml, 72 h) treatment (n = 3, with independent sample *t* test). **F**, immunoblot analysis was used to detect the expression of NF-κB related biomarkers in LN229 and U87 cells following FOSL1 knock-down, with or without TNFα (200 ng/ml, 72 h) treatment. GAPDH was used as the loading control for normalization. **P*<0.05, ***P*<0.01, ****P*<0.001,

****P<0.0001. Data shown as mean ± SD. The immunoblotting experiments were repeated three times with similar results.

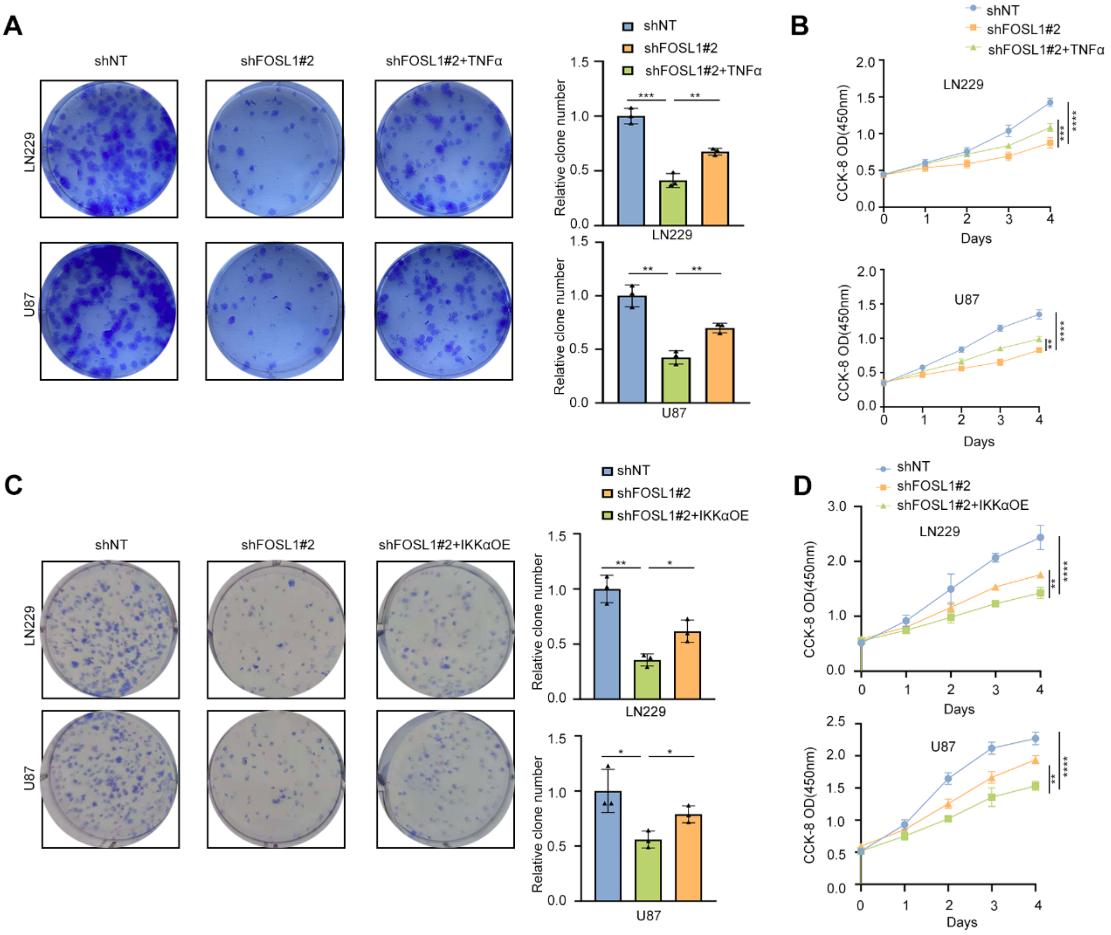

Figure S7, related to Figure 2. FOSL1 promoted malignancies of GBM through activation of NF-κB signaling pathway. A and B, Colony formation (n = 3, analyzed by independent sample *t* test) and CCK-8 (n = 3, with one-way ANOVA test) assays were conducted to assess cell proliferation in LN229 and U87 cells following FOSL1 knock-down, with or without TNFα (200 ng/ml, 72 h) treatment. **C and D,** Colony formation (n = 3, analyzed by independent sample *t* test) and CCK-8 (n = 3, with one-way ANOVA test) assays were conducted to assess cell proliferation in LN229 and U87 cells following FOSL1 knock-down, with or without IKKα overexpression. *$P < 0.05$, **$P < 0.01$, ***$P < 0.001$, ****$P < 0.0001$. Data shown as mean ± SD.

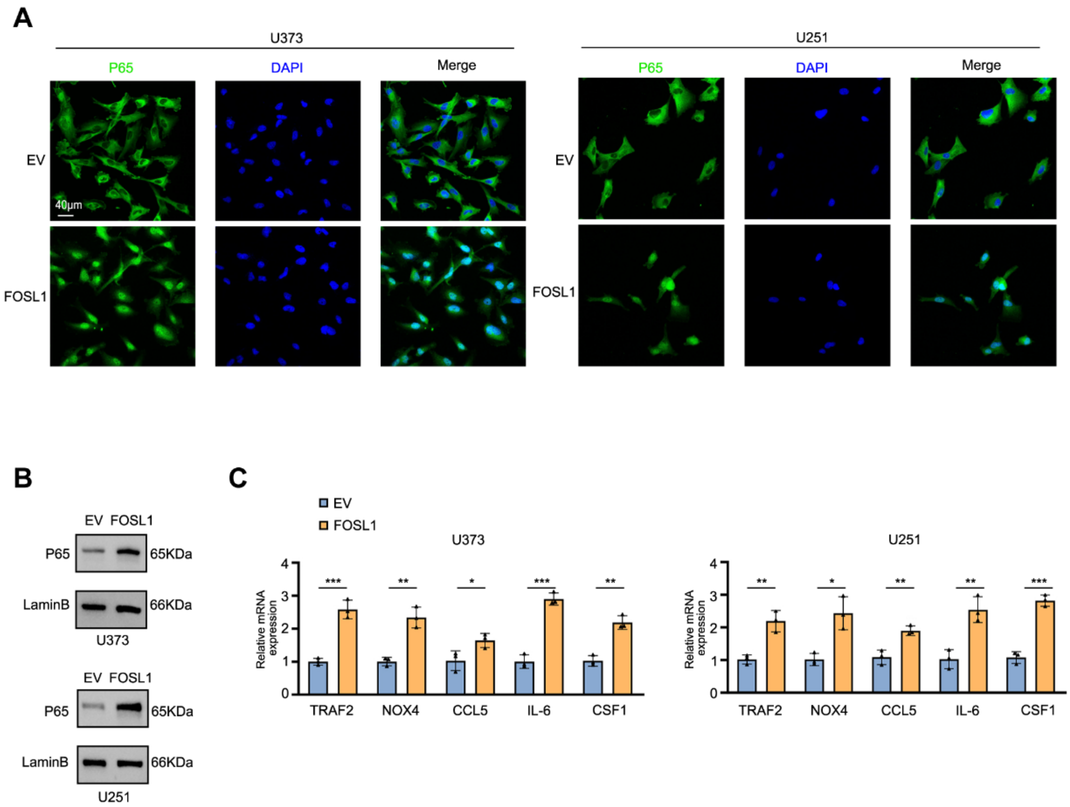

Figure S8, related to Figure 2. FOSL1 promoted malignancies of GBM through activation of NF-κB signaling pathway. A, Representative image of the P65 (green) protein in U373 (Left) and U251 (Right) cells observed by confocal microscope. Scale bars, 40 μm. **B,** immunoblot analysis was used to detect the expression of P65 related biomarkers in U373 and U251 cells following FOSL1 overexpression. LaminB was used as the loading control for normalization. **C,** qRT-PCR assays for expression of NF-κB signaling correlated downstream targets in U373 and U251 cells following FOSL1 overexpression. $*P < 0.05$, $**P < 0.01$, $***P < 0.001$. Data shown as mean ± SD. The immunoblotting experiments were repeated three times with similar results.

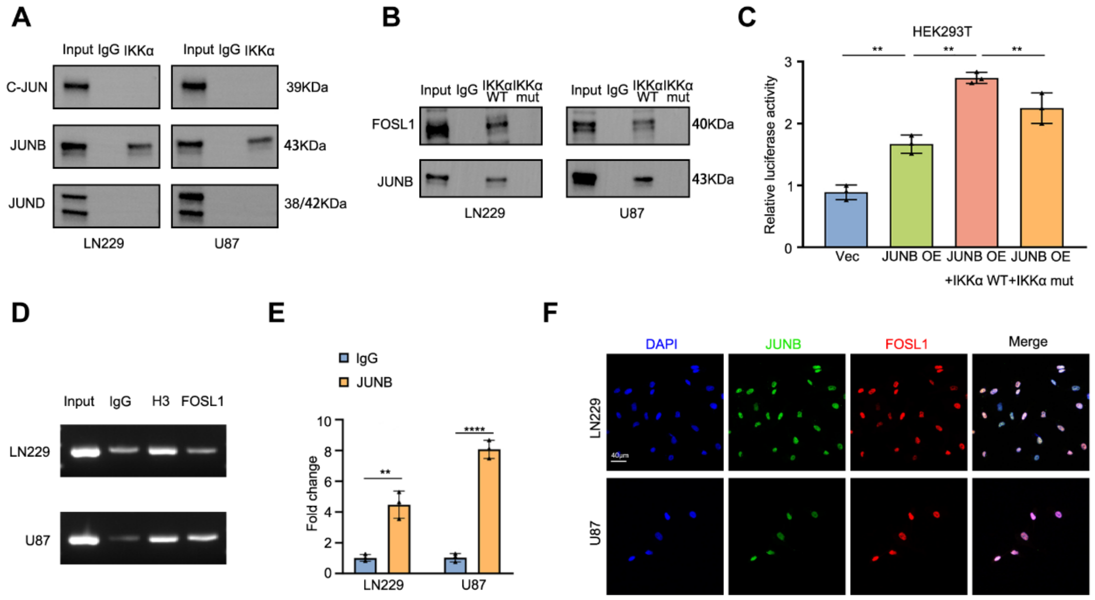

Figure S9. JUNB transcriptionally up-regulated *IKKα* expression in GBM. A and B, Biotin-labeled DNA fragments containing either wild-type or mutant *IKKα* promoter sequences were immobilized on streptavidin-coated magnetic beads and incubated with nuclear extracts from glioblastoma (GBM) cells. The captured protein complexes were subsequently analyzed by immunoblot. **C,** the direct activation of *IKKα* by JUNB was validated by the luciferase activity assay (n = 3, analyzed by independent sample *t* test). **D and E,** ChIP PCR and qRT-PCR (n = 3, analyzed by independent sample *t* test) analysis of JUNB binding to the *IKKα* promoter in LN229 and U87 cells. **F,** Representative image of the co-localization of FOSL1 (red) and JUNB (green) protein in LN229 (upper) and U87 (bottom) cells observed by confocal microscope. GAPDH was used as the loading control for normalization. Scale bars, 100 μm. Ns = not significant, **P* < 0.05, ***P* < 0.01, ****P* < 0.001, *****P* < 0.0001. Data shown as mean ± SD. The immunoblotting experiments were repeated three times with similar results.

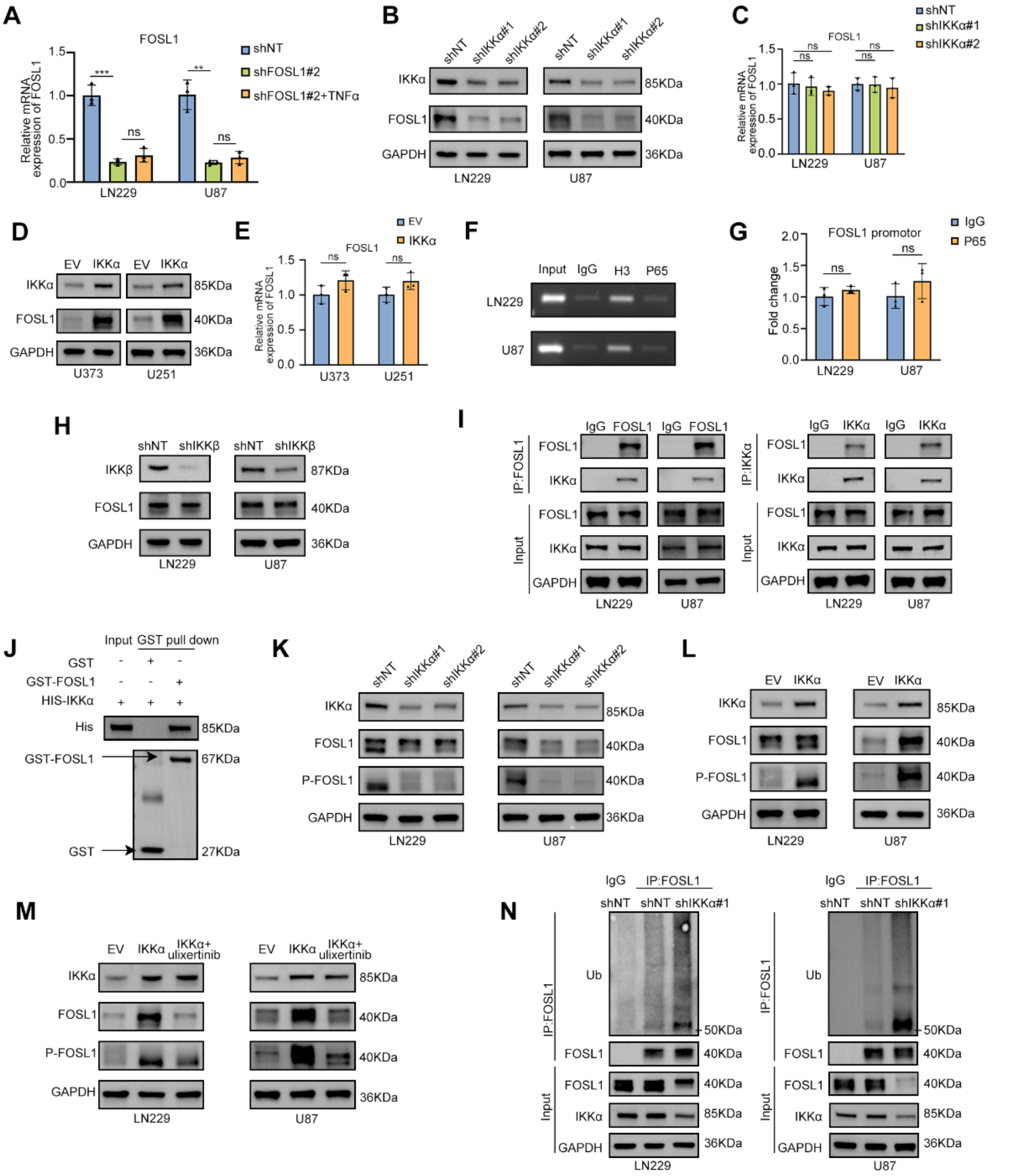

Figure S10, related to Figure 4. IKKα phosphorated FOSL1 thus enhanced the stability of FOSL1.

A, qRT-PCR (n = 3, with independent sample *t* test) was used to detect the expression of FOSL1 in LN229 and U87 cells following FOSL1 knock-down, with or without TNFα (200 ng/ml, 72 h) treatment. **B and C,** qRT-PCR (n = 3, with independent sample *t* test) and immunoblot analysis was used to detect the expression of FOSL1 in LN229 and U87 cells following IKKα knock-down. **D and E,** the expression of FOSL1 was confirmed by qPCR (n = 3, with independent sample *t* test) and Immunoblot analysis in U373 and U251 following IKKα overexpression. **F and G,** ChIP-PCR and qPCR (n = 3, analyzed by independent sample *t* test) analysis of P65 binding to the FOSL1 promoter in LN229 and U87 cells. **H,** immunoblot analysis was used to detect the expression of FOSL1 in LN229 and U87 cells following IKKβ knock-down. **I,** immunoblot analysis of endogenous FOSL1 and IKKα

expression in a co-IP assay performed in LN229 and U87 cells with protein A/G magnetic beads and anti-FOSL1 (H) or anti-IKKα (I) primary antibody. **J,** the physical interaction between FOSL1 and IKKα was confirmed by GST pull-down assays. GST protein alone served as the negative control. **K,** immunoblot analysis was used to detect the phosphorylation status of FOSL1 in LN229 and U87 cells following IKKα knock-down. **L,** the phosphorylation status of FOSL1 was confirmed immunoblot analysis in U373 and U251 following IKKα overexpression. **M,** The expression and phosphorylation status of FOSL1 was confirmed immunoblot analysis in U373 and U251 following IKKα overexpression with or without an ERK2 inhibitor, ulixertinib (10 μM, 24 h). **N,** LN229 and U87 cells with or without IKKα knock-down, followed by immunoprecipitation with anti-FOSL1 primary antibody. GAPDH was used as the loading control for normalization. Ns = not significant, **P < 0.01, ***P < 0.001. Data shown as mean ± SD. The immunoblotting experiments were repeated three times with similar results.

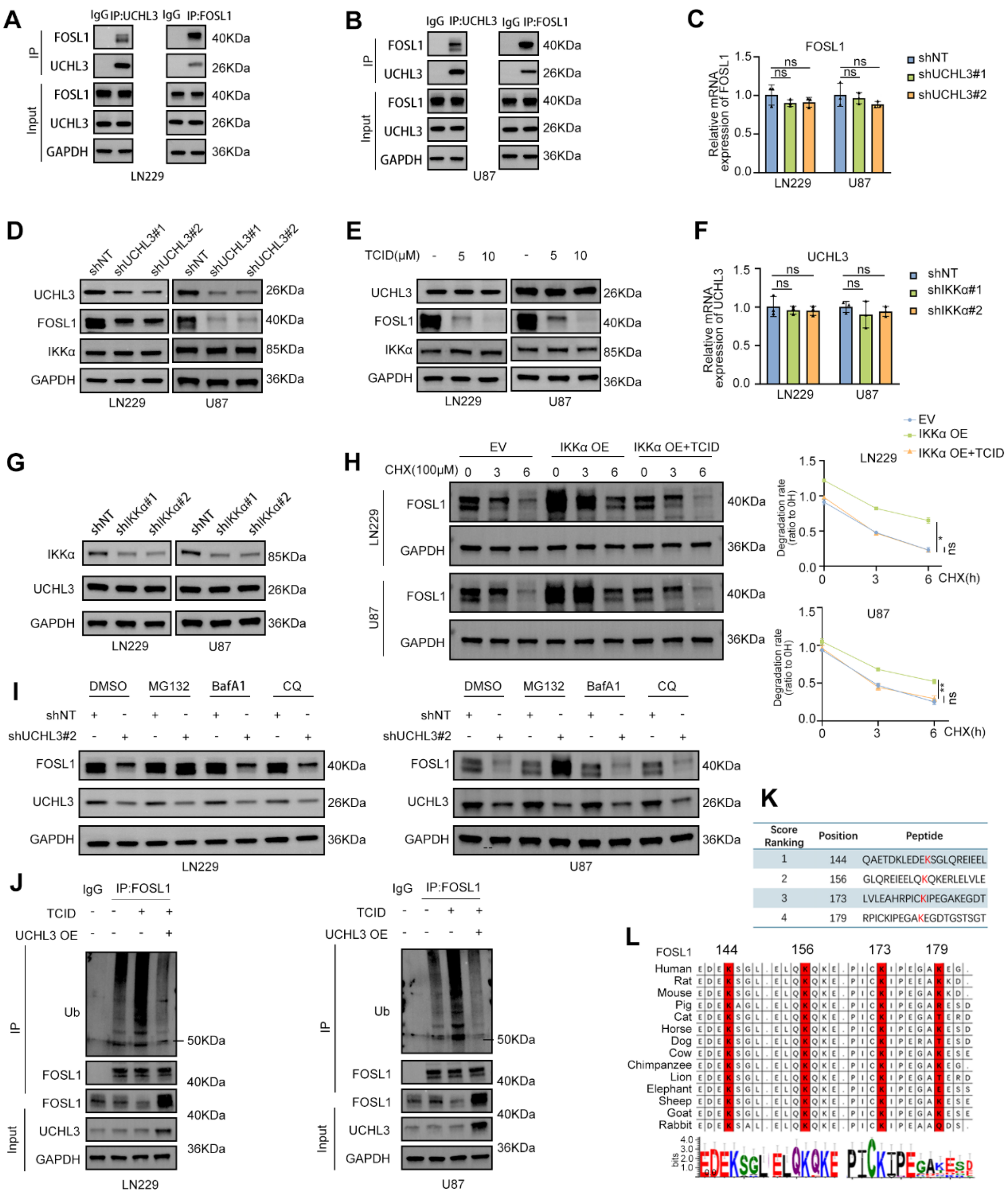

Figure S11, related to Figure 5. UCHL3 was essential for IKKα-mediated stabilization of FOSL1. A and B, Co-IP assays were performed in LN229 (A) and U87 (B) cells using Protein A/G Magnetic Beads with anti-UCHL3 (left) or anti-FOSL1 (right) primary antibodies, follow by immunoblot analysis. **C and D,** qRT-PCR (n = 3, analyzed by independent sample *t* test) and immunoblot analysis was used to detect the expression of FOSL1 and IKKα in LN229 and U87 cells following UCHL3 knock-down. **E,** Immunoblot analysis was used to detect the expression of FOSL1 and IKKα in LN229 and U87 cells treated with or without UCHL3 inhibitor TCID (10 μM, 24 h). **F and G,** qRT-PCR (n = 3, analyzed by independent sample *t* test) and immunoblot analysis was used to detect the expression of UCHL3 in LN229 and U87 cells following IKKα knock-down. **H,** LN229 and U87 cells with or without IKKα knock-down and UCHL3 inhibitor TCID (10 μM, 24 h),

followed by cycloheximide (CHX; 100 μM) for 0, 3, 6 h were used for immunoblot analysis to measure the protein levels of FOSL1. Density of FOSL1 expression was quantified by ImageJ. **I,** LN229 and U87 cells with or without UCHL3 knock-down were treated with the proteasome inhibitor MG132 (25 μM, 8 h), autophagy inhibitor bafilomycin A1 (BAFA1; 30 nM, 2 h), or chloroquine (CQ; 30 μM, 30 min). FOSL1 and UCHL3 levels were analyzed by immunoblot. **J,** UCHL3 decreases ubiquitination of FOSL1. LN229 and U87 cells with or without UCHL3 overexpression were treated with a UCHL3 inhibitor TCID (10 μM，24 h), followed by immunoprecipitation with anti-Flag primary antibody and immunoblot analysis. **K,** Ubiquitination sites predicted by GPS Uber. **L,** Sequence conservation analysis of relevant amino acids of FOSL1. GAPDH was used as the loading control for normalization. Ns = not significant. Data shown as mean ± SD. The immunoblotting experiments were repeated three times with similar results.

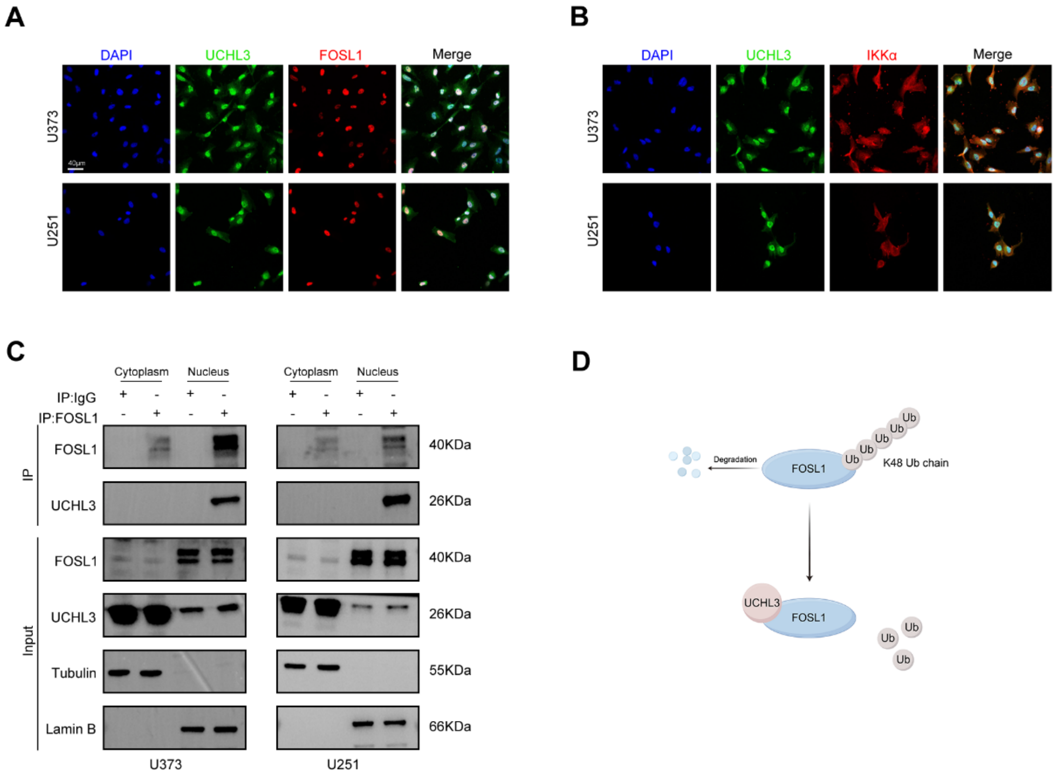

Figure S12, related to Figure 5. UCHL3 was essential for IKKα-mediated stabilization of FOSL1. A, Representative image of the co-localization of FOSL1 (red) and UCHL3 (green) protein in LN229 (upper) and U87 (bottom) cells observed by confocal microscope. Scale bars, 40 μm. **B,** Representative image of the co-localization of IKKα (red) and UCHL3 (green) protein in LN229 (upper) and U87 (bottom) cells observed by confocal microscope. **C,** Subcellular fractionation followed by co-IP assay was performed to investigate FOSL1-UCHL3 interactions in LN229 (left) and U87 (right) cells. Tubulin and Lamin B served as cytoplasmic and nuclear loading controls, respectively, for normalization. The immunoblotting experiments were repeated three times with similar results. **D,** Schematic representation for the UCHL3 targeted the K48-linked polyubiquitin chain of FOSL1.

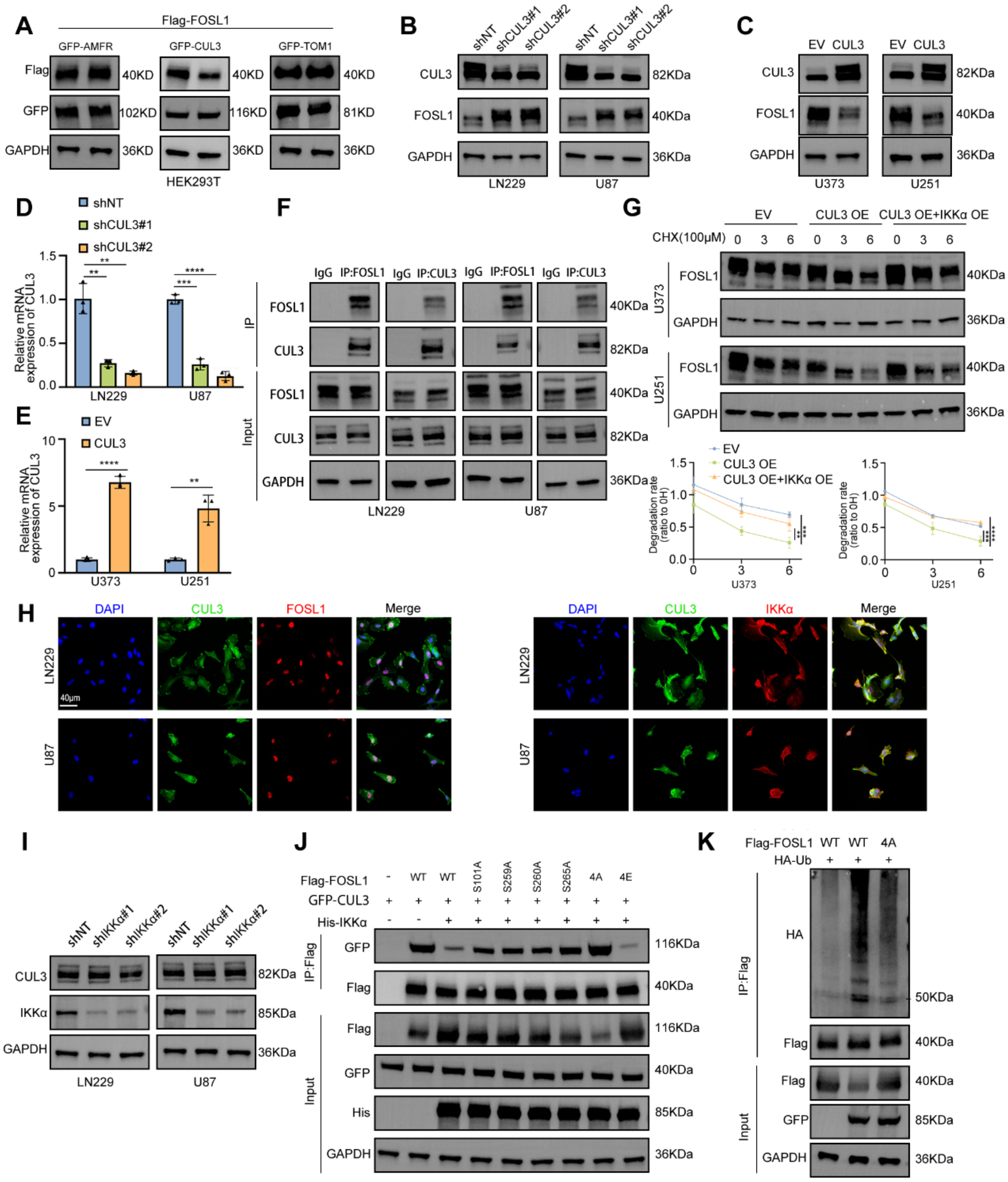

Figure S13, CUL3 acts as an E3 ubiquitin ligase for FOSL1. A, FOSL1 protein levels were assessed in HEK293T cells overexpressing GFP-tagged DUB candidates (CUL3, TOM1, and AMFR) alongside Flag-FOSL1. **B-E,** qRT-PCR (n = 3, with independent sample *t* test) and immunoblot analysis was used to detect the expression of FOSL1 in LN229 and U87 cells following CUL3 knock-down or overexpression. **F,** Co-IP assays were performed in LN229 and U87 cells using Protein A/G Magnetic Beads with anti-FOSL1 (left) or anti-CUL3 (right) primary antibodies, follow by immunoblot analysis. **G,** LN229 and U87 cells with or without CUL3 overexpression, followed by cycloheximide (CHX; 100 μM) for 0, 3, 6 h. Density of FOSL1 expression was quantified by ImageJ. **H,** Representative image of the co-localization of FOSL1 (red) and CUL3 (green) protein (Left) and IKKα (red) and CUL3 (green) protein (Right) in LN229 (upper) and U87 (bottom) cells observed by confocal

microscope. Scale bars, 40 μm. **I,** immunoblot analysis was used to detect the expression of IKKα in LN229 and U87 cells following CUL3 knock-down. **I,** HEK293T cells were transfected with Flag-FOSL1, various HA-ubiquitin mutants, GFP-CUL3 and His-IKKα. **O,** HEK293T cells were transfected with or without FOSL1 mutant plasmids, followed by immunoprecipitation with anti-Flag primary antibody. GAPDH was used as the loading control for normalization. $^{**}P < 0.01$, $^{***}P < 0.001$, $^{****}P < 0.0001$. Data shown as mean ± SD. The immunoblotting experiments were repeated three times with similar results.

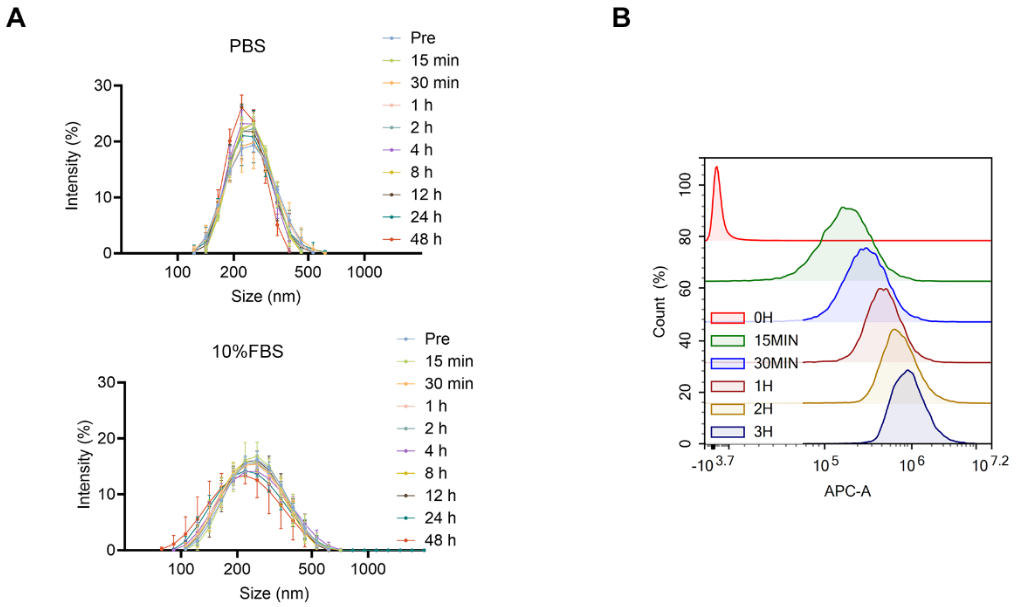

Figure S14, related to Figure 6 Development and evaluation of a nanocapsuled siRNA delivery system for GBM therapy. A, The stability of plofsome@*siFOSL1* in PBS and 10% FBS for 48 h. B, Flow cytometry analysis of the cellular uptake of U87 cells treated with plofsome@*siNC*/APC.

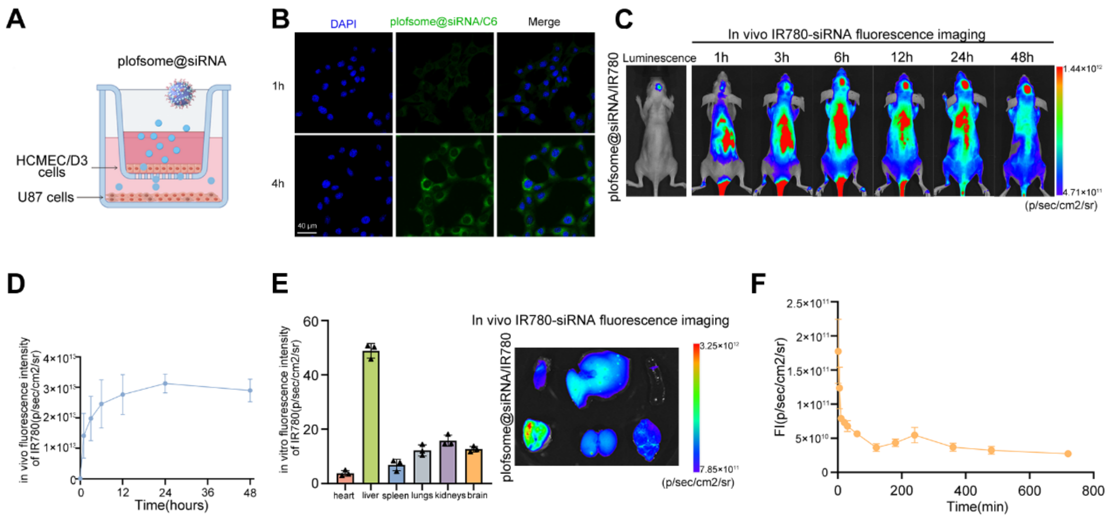

Figure S15, related to Figure 7 ***In Vivo*** **evaluation of plofsome@*siFOSL1* for GBM therapy. A,** Schematic diagram of the BBB model *in vitro*. **B,** plofsome@*siNC*/C6 across the BBB in an *in vitro* transwell BBB model. Representative image in different hours of the plofsome@*siNC*/C6 in U87 cells observed by confocal microscope. Scale bars, 40 μm. **C,** *In vivo* fluorescence images of tumor-bearing nude mice at indicated times post-i.v. injection of plofsome@*siNC*/IR780. **D,** the intensity of intracranial fluorescence was quantified using IVIS image system (n = 3). **E,** the fluorescence intensity in major organs of nude mice was quantified using IVIS image system (n = 3). F, In *vivo* pharmacokinetics of plofsome@*siNC*/IR780 in tumor-free mice.

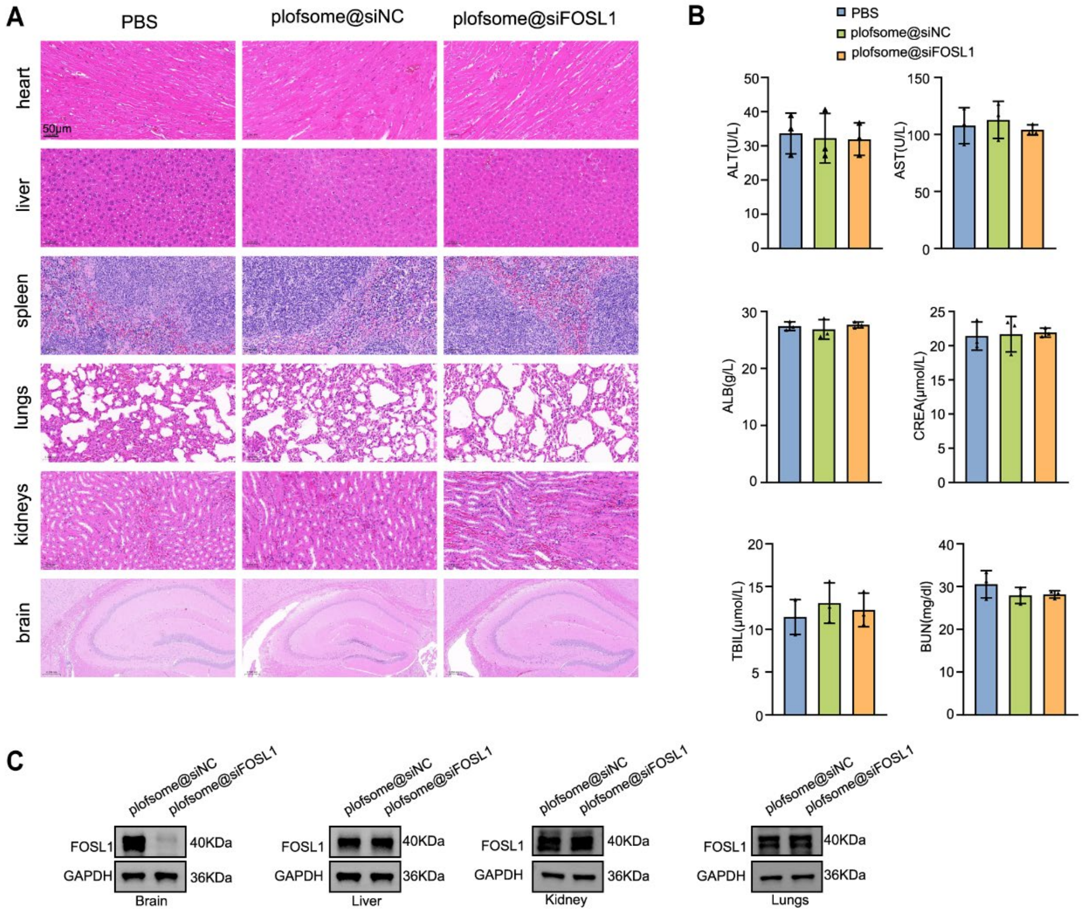

Figure S16, Biosafety evaluation of plofsome@*siFOSL1*. A, Histological analyses of the major organs (heart, liver, spleen, lung, kidney, and non-tumor brain regions) from tumor-bearing nude mice following treatment with PBS, plofsome@siNNC and plofsome@*siFOSL1*. Scale bars, 50 μm. **B,** Biochemical index analysis of ALT, AST, ALB, TBIL, BUN, and CREA. Data are shown as the mean ± SD (n = 3). C, Immunoblot analysis of FOSL1 protein levels in tumor, liver, kidney and lung tissues after plofsome@*siFOSL1* treatment. GAPDH was used as the loading control for normalization. Ns = not significant. Data shown as mean ± SD. The immunoblotting experiments were repeated three times with similar results.

Supplementary Tables

Table S1 The information of primary antibodies

Antibodies	Source	Cat#
Anti-FOSL1	Abcam	ab124722
Anti-FOSL1 (IP)	Santa Cruz	sc-28310
Anti-IKKα	Proteintech	84372-5-RR
Anti-IKKα (IP)	Santa Cruz	sc-7606
Anti-P-IKKα (phospho T23)	Abcam	ab38515
Anti-IκBα	Zenbio	R23322
Anti-NFκB	CellsignalingTechnology	#8242
Anti-P-NFκB (Ser536)	CellsignalingTechnology	#3033
Anti-IKKβ	Proteintech	15649-1-AP
Anti-Cullin 3	Abways	CY7196
Anti-Cullin 3(IP)	Abways	sc-166110
Anti-H3	CellsignalingTechnology	#4620
Anti-Rabbit IgG	CellsignalingTechnology	#2729
Anti-IgG-R	Proteintech	B900620
Anti-Flag	Proteintech	20543-1-AP
Anti-Flag (IP)	Santa Cruz	sc-166355
Anti-HA	Proteintech	51064-2-AP
Anti-Myc	Proteintech	10828-1-AP
Anti-His	Proteintech	84814-1-RR
Anti-His (IP)	Santa Cruz	sc-8036
Anti-Tubulin	Zenbio	R23452
Anti-Lamin B	Santa Cruz	sc-56144
Anti-GFP	Proteintech	50430-2-AP
Anti-GFP (IP)	Santa Cruz	sc-9996
Anti-GST	Proteintech	81527-1-RR
Anti-GST (IP)	Santa Cruz	sc-138
Anti-P-FOSL1 (Ser265)	CellsignalingTechnology	#5841
Anti- Ubiquitin	Proteintech	10201-2-AP
Anti-UCHL3	Zenbio	R380869
Anti-UCHL3 (IP)	Santa Cruz	sc-100340
Anti-GAPDH	Proteintech	HRP-60004

Table S2 Primers for qRT PCR

Primers	Sequences-F (5' → 3')	Sequences-R (5' → 3')
FOSL1	ACTGGAAGATGAGAAATCTGGG	GGGAAAGGGAGATACAAGGTAC
IKKα	ATGAAGAAGTTGAACCATGCCA	CCTCCAGAACAGTATTCCATTGC
IKKβ	CTGGCCTTTGAGTGCATCAC	CGCTAACAACAATGTCCACCT
UCHL3	AGAACGAGCCAGATACCTGGA	GCTTCCGCCCATCTAATTCAT
GAPDH	AGAAGGCTGGGGCTCATTTG	AGGGGCCATCCACAGTCTTC

Table S3 Primers for ChIP PCR and qRT PCR

Primers	Sequences-F (5' → 3')	Sequences-R (5' → 3')
FOSL1	TCCCCGAAGTCTCGGAACAT	TGGTTCAGCCCGAGAACTTT
IKKα	CTCGCGAGAATGAATGCGTC	CATTGTGGTTCCGTTCAGCC

Table S4 Sequences for gene knockdown

siFOSL1	CCAGCCUGGUCUUCACCUA	UAGGUGAAGACCAGGCUGG
siUCHL3	GGAUUGUUGUGAAGACUAAUG	UUAGUCUUCACAACAAUCCCA
shFOSL1#1	CCAAGCATCAACACCATGAGT	
shFOSL1#2	CTGTACCTTGTATCTCCCTTT	
shIKKα#1	GCAAATGAGGAACAGGGCAAT	
shIKKα#2	GCGTGCCATTGATCTATATAA	
shIKKβ	GCACTGGGAAAGTATCTGAAA	
shUCHL3#1	GCACCAAGTATAGATGAGAAA	
shUCHL3#2	CCTGGAGGAATCTGTGTCAAT	
ShCUL3#1	CGTGTGCCAAATGGTTTGAAA	
ShCUL3#2	CGTAAGAATAACAGTGGTCTT	
